# Supplementary material for: Semi-conducting 2D rectangles with tunable length via uniaxial living crystallization-driven self-assembly of homopolymer
Source: Nat Commun. 2021 May 10;12:2602. doi: 10.1038/s41467-021-22879-6 (PMC8110585; doi:10.1038/s41467-021-22879-6)
Supplement: Supplementary file 1 — Supplementary Information [file 41467_2021_22879_MOESM1_ESM.pdf]

Supplementary Information

Semi-conducting 2D Rectangles with Tunable Length via Uniaxial Living Crystallization-Driven Self-Assembly of Homopolymer

Sanghee Yang[1], Sung-Yun Kang[1], and Tae-Lim Choi*[1]

[1]*Department of Chemistry, Seoul National University, Seoul 08826, Republic of Korea*

Email: tlc@snu.ac.kr

Table of Contents

1. General Analytic Methods S2
2. General Materials and Polymers S3
3. Supporting Figures and Tables S4–S33
4. Supplementary References S34

1. General Analytic Methods

Characterization of substrates and polymers. NMR spectra were recorded by Varian/Oxford As-500 (500 MHz for ^1H and 125 MHz for ^{13}C) spectrometer and Agilent 400-MR (400 MHz for ^1H and 100 MHz for ^{13}C). Size exclusion chromatography (SEC) analyses were carried out with the Waters system (a 515 pump and a 2707 autosampler with a loop volume of 100*m*L), Wyatt OptiLab T-rEx refractive index detector and Shodex SEC LF-804 column eluted with chloroform (SEC grade, Honeywell Burdick & Jackson). The flow rate was 1.0 mL/min and the temperature of the column was maintained at 35 °C. Samples were diluted in 1 g/L by chloroform and filtered through a 0.20 mm PTFE filter before using.

Characterization of nanostructures. Dynamic light scattering (DLS) data was obtained with a polymer solution (in 1 g/L in general) in quartz glass cell (Hellma Analytics) by Malvern Zetasizer Nano-S. UV-vis spectra were obtained by Jasco Inc (UV-vis spectrometer V-650). For atomic force microscopy (AFM) imaging, Multimode 8 and Nanoscope V controller (Veeco Instrument) was used. Tapping with non-contact mode was used to obtain AFM images and AFM tips are commercially available from Nanoworld (Pointprobe® tip, NCHR type, a spring constant of 42 N/m and a radius of tip ≤ 8 nm.) Transmission electron microscopy (TEM) imaging was performed by using JEM-2100 (JEOL) at 120 kV. The cryo-TEM analysis was carried out by using the same microscope. Carl Zeiss LSM710 was used for laser scanning confocal microscopy (LSCM) with 488 and 543 nm excitation. Fluorescent image from 561 nm excitation was obtained with SP8 X STED laser from normal LSCM. More advanced super-resolution optical microscope images were obtained from ELYRA PS.1 in the National Center for Inter-University Research Facility at SNU.

Statistical analysis of the size of nanostructures. For each nanostructure, length, area, aspect ratio, and angle distributions were estimated from the AFM and TEM images manually using the ImageJ software package which developed at the US National Institute of Health. For the statistical length analyses, more than 30 of randomly picked objects were processed to determine the average values depending on the data set. Every particle in each image was counted to reduce subjectivity. From this data, histogram were constructed and values of the number average contour length (L_n), width (W_n), area (A_n) standard deviation (s) and polydispersity index ($Đ$) were estimated using the following equations where N is the sample size:[1]

$$L_\mathrm{n} = \frac{\sum_{i=1}^{n} N_i L_i}{\sum_{i=1}^{n} N_i} \qquad L_\mathrm{w} = \frac{\sum_{i=1}^{n} N_i L_i^2}{\sum_{i=1}^{n} N_i L_i}$$

$$A_\mathrm{n} = \frac{\sum_{i=1}^{n} N_i A_i}{\sum_{i=1}^{n} N_i} \qquad A_\mathrm{w} = \frac{\sum_{i=1}^{n} N_i A_i^2}{\sum_{i=1}^{n} N_i A_i}$$

$$\sigma = \sqrt{\frac{1}{N}\sum_{i=1}^{n}(x_i - \mu)^2}$$

Kinetic data fitting. Fitting of the kinetic data was performed using Origin (OriginLab, Northampton, MA) software. For the growth kinetic studies with one set of 2D seeds, the seed size was fixed to the value determined by analysis of the TEM contour lengths and areas for all the sets. The standard deviation of the size factors at each time point was used to weight the fits (instrumental error). The values were obtained when the fit converged and R^2 was higher than 0.99.

2. General Materials and Polymers

Materials: Without additional notes, all reagents were commercially available from Sigma-Aldrich®, Tokyo Chemical Industry Co. Ltd., Acros Organics, Alfa Aesar® and were used without further purification. Solvents for monomer synthesis were also commercially available, but they for polymerization are used after further-distillation under $Ar_{(g)}$. All the reactions were conducted under the $Ar_{(g)}$, and monitored by thin-layer chromatography carried out on pre-coated plates (MERCK TLC silica gel 60, F_{254}). For purification, flash column chromatography was performed using MERCK silica gel 60 (0.040 ~ 0.063 mm). For polymerization, the Grubbs third-generation (G3) catalyst was prepared following the reported literature.[2]

Polymers

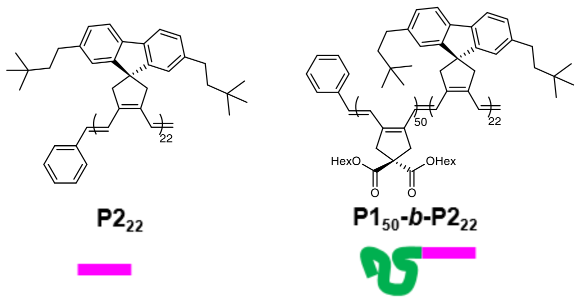

Sample	Block ratio	M_n (kDa)	$Đ$
**P1**$_{50}$-*b*-**P2**$_{22}$	50:22	38.6	1.10
**P2**$_{22}$	-	9.01	1.13
**P2**$_{10}$	-	4.98	1.15
**P2**$_{13}$	-	5.61	1.18
**P2**$_{15}$	-	6.05	1.13

3. Supplementary Figures. and Tables

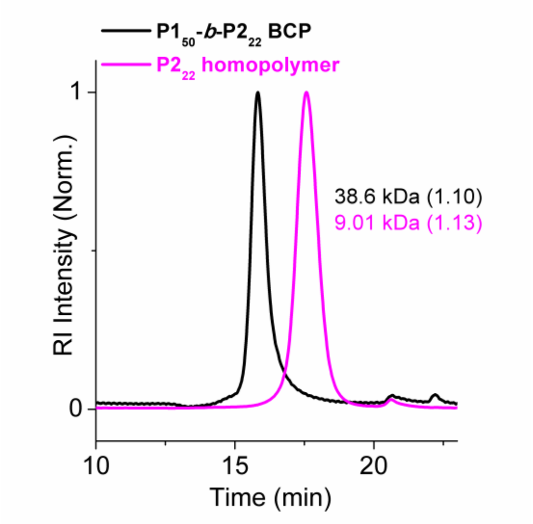

Supplementary Fig. 1 Normalized chloroform SEC traces of P1$_{50}$-*b*-P2$_{22}$ BCP (M_n = 38.6 kDa ($Đ$ = 1.10)) and P2$_{22}$ homopolymer (M_n = 9.01 kDa ($Đ$ = 1.13)).

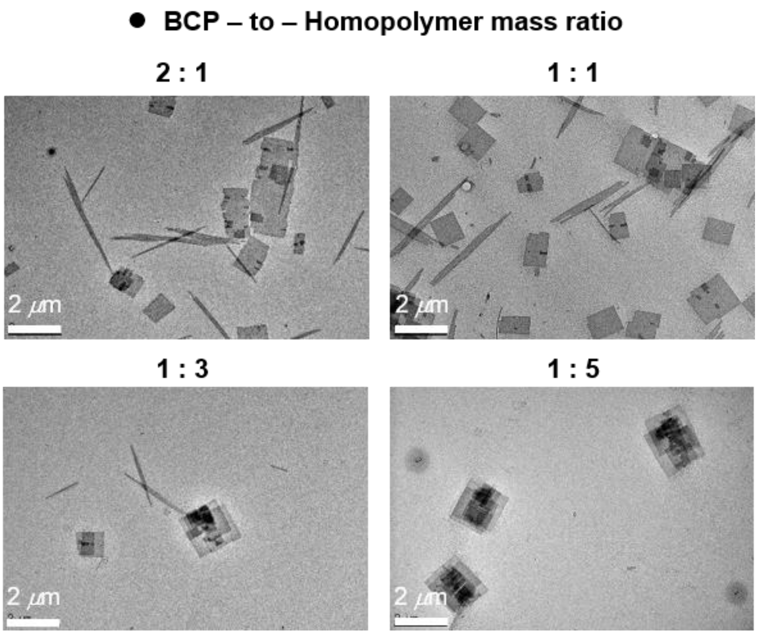

Supplementary Fig. 2 TEM images of the blends with various mass ratios of the P1$_{50}$-*b*-P2$_{22}$ BCP and P2$_{22}$ homopolymer in 1 g/L chloroform after 1 hour heating at 60 °C followed by aging at 25 °C for 12 hours.[3] As shown in Fig. 1a, multi-stacked 2D rectangles of the homopolymer[4] and 1D nanofibers of BCP[5] have been reported. In the growth mechanisms of the multi-stacked 2D rectangles, P2 homopolymer assembles in both the (100) and (010) directions of the crystalline plane, while BCP crystallizes first in the (100) direction, resulting in the micrometer-long 1D nanofibers. Therefore, as the mass of the BCP increases during co-assembly, more 1D ribbon-like structures appear close to the 1D nanofibers of BCP. Conversely, when the portions of P2 homopolymer increases in the blend, 2D nanosheets from the P2 homopolymer are created, but with suppression of the multi-stack due to the BCP minimizing the interaction between each sheet. If the portions of P2 homopolymer becomes too high, then the formation of multi-stacked 2D nanosheets is dominant again.

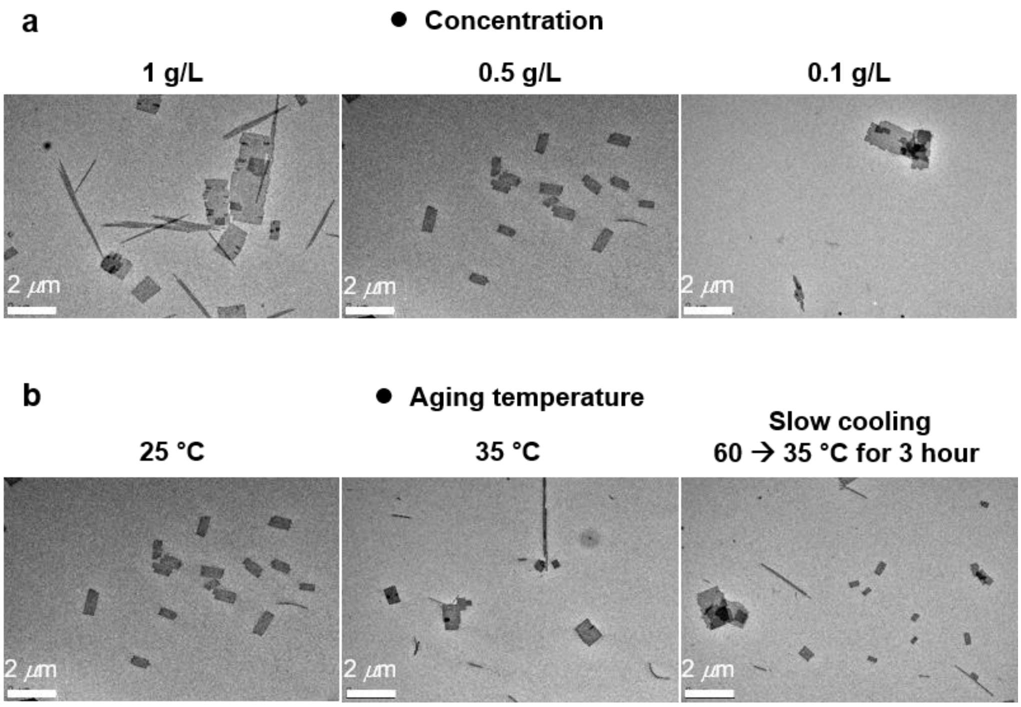

Supplementary Fig. 3 a, TEM images of the blends with the BCP and Homopolymer mass ratio of 2 at various concentrations in chloroform after 1 hour heating at 60 °C followed by aging at 25 °C for 12 hours. The 0.5 g/L was found to be the optimal concentration for the co-assembly of two polymers. **b,** TEM images of the blends with the mass ratio of 2 in 0.5 g/L chloroform after 1 hour heating at 60 °C followed by aging at various temperatures for 12 hours. Conclusively, 25 °C was optimal aging temperature. All the experiments were performed with the blends in 4 mL vial.

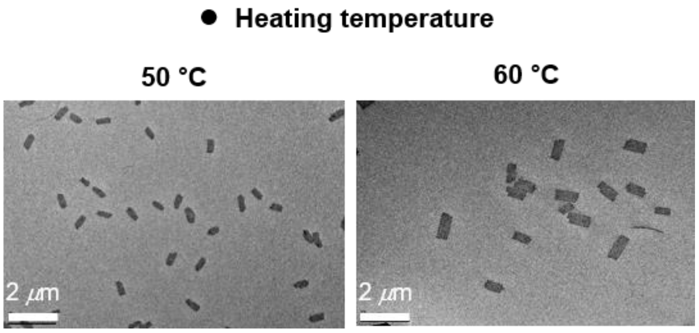

Supplementary Fig. 4 TEM images of the blend with the mass ratio of 2 in 0.5 g/L chloroform after 1 hour heating at various temperatures followed by aging at 25 °C for 12 hours. By heating at 50 °C, more uniform 2D nanosheets were produced via co-assembly without any 1D defect.

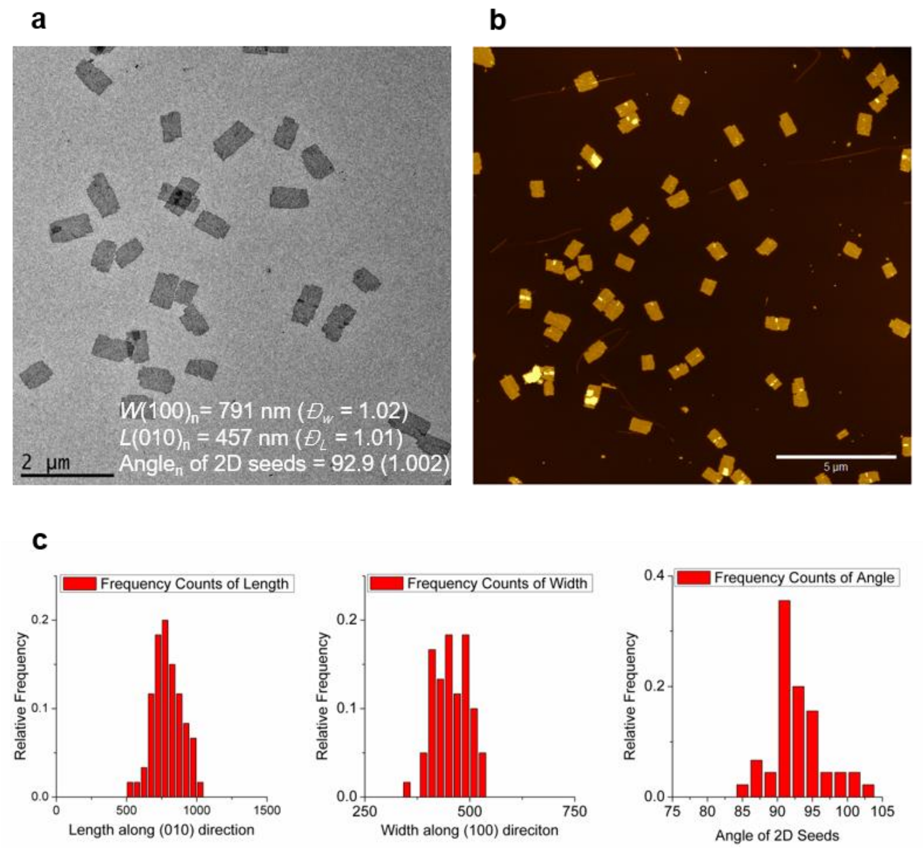

Supplementary Fig. 5 Low magnified TEM image (**a**) and AFM image (**b**) of the uniform mono-layered 2D rectangles prepared by the aforementioned optimal condition ($P1_{50}$-*b*-$P2_{22}$ – to – $P2_{22}$ mass ratio of 2:1 (mole ratio of 1:2) in 0.5 g/L chloroform was heated for 1 hour at 50 °C and then aged for 3 days at 25 °C). Numbers in the images indicate the average W_n, L_n, and $angle_n$ and their length (or angle) dispersities. **c,** Length, width, and angle histograms of the 2D nanosheets obtained from randomly picked 50 samples.

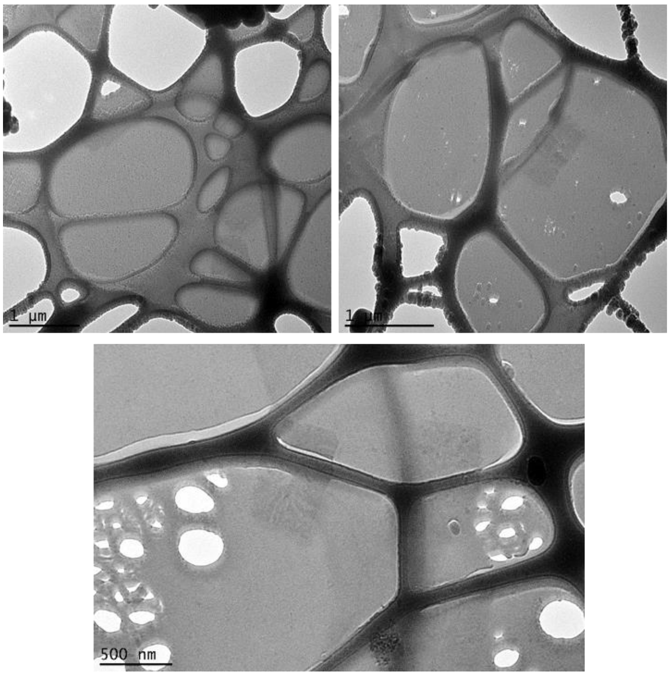

Supplementary Fig. 6 Cryogenic TEM images of the 2D seeds by freezing the 0.05 g/L in chloroform (after dilution from 0.5 g/L seed solution in chloroform). This confirmed that the co-asembly to the 2D seeds occurred in solution (not by solvent evaporation).

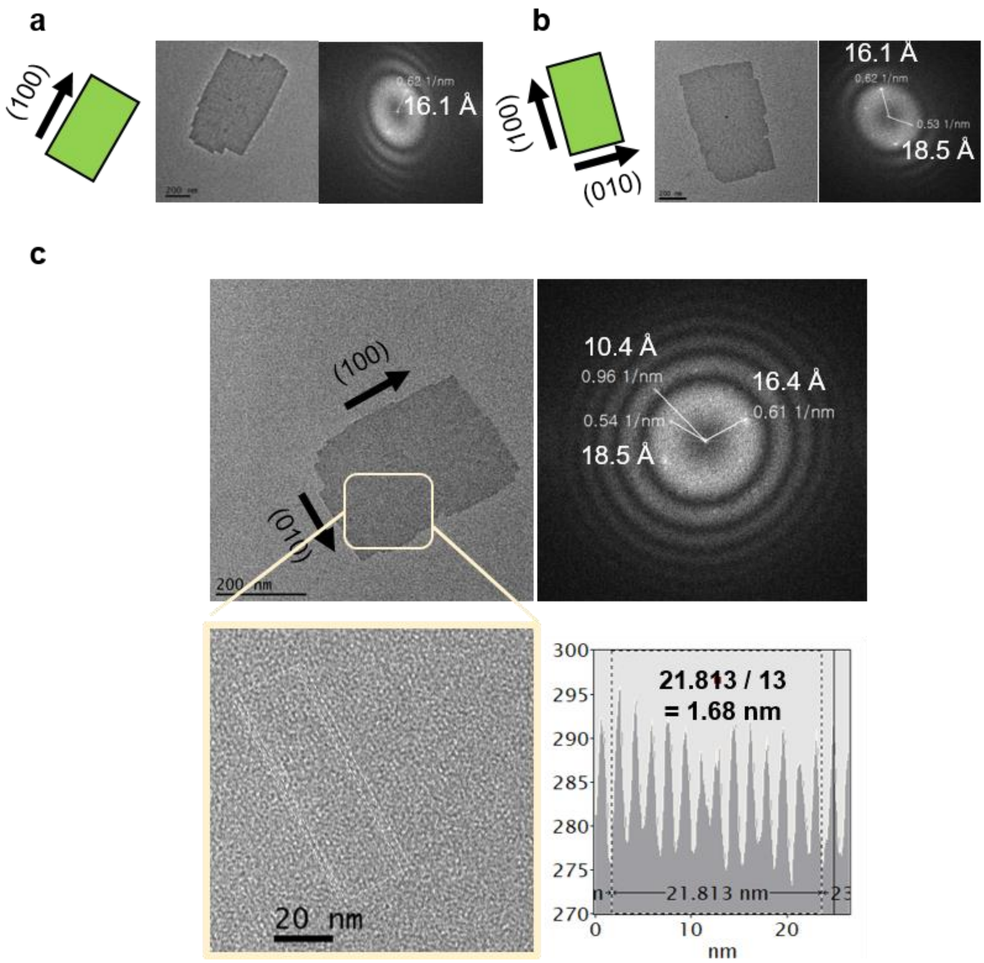

Supplementary Fig. 7 a-c, HR-TEM images of the 2D seeds with their FFT patterns showing *d*-spacing of 16.1–16.4 Å. Based on our previous findings on the orthorhombic crystal lattice of the P2 homopolymer, we assigned the 16.1 Å as a *d*-spacing in the (200) plane.[4] In **Supplementary Fig. 7c**, we offered an additional cross-sectional histogram of one 2D seed with an electron density profile; *d*-spacing of 16.8 Å was also calculated from the electron density difference.

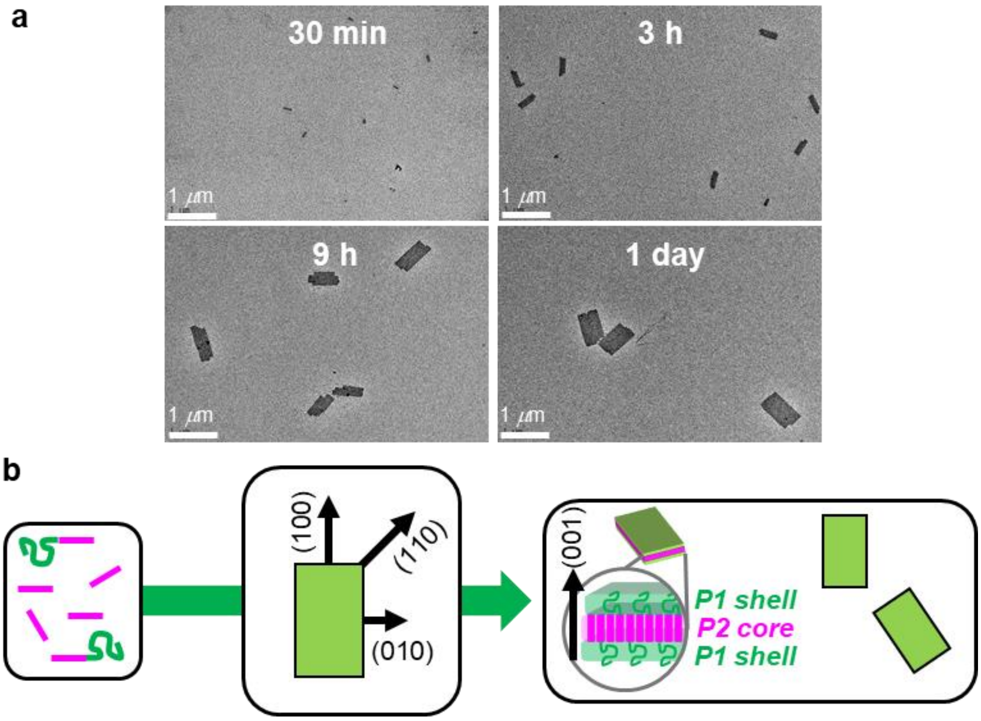

Supplementary Fig. 8 a, The formation process of 2D seeds over aging time from 30 min (after heating) to 1 day. **b,** Schematic illustration of the 2D seeds formation with different crystal growth rates. From their rectangular shapes, we could infer that the different width and length values would be due to the difference in crystal growth rate of each plane. Thus, one side (100) of the rectangular nanosheets would grow faster than the other side (010), leading to the formation of an anisotropic rectangular nanosheets.[3]

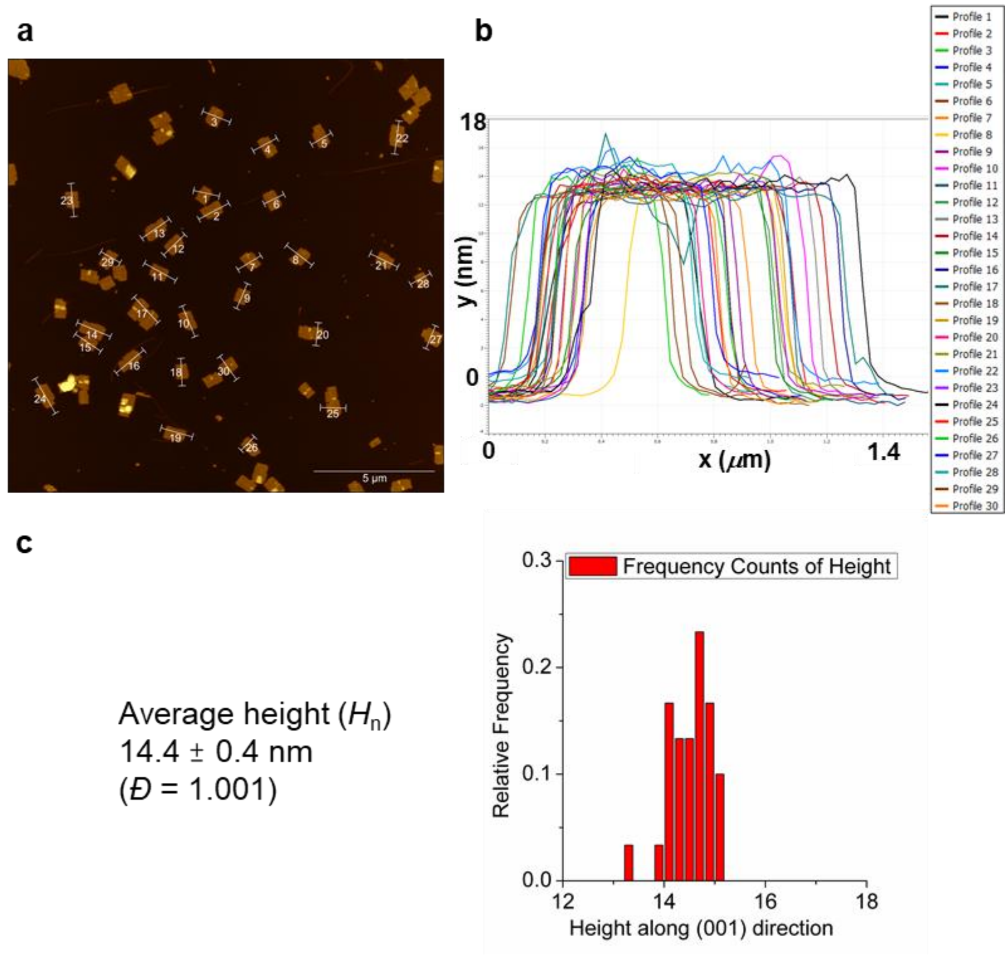

Supplementary Fig. 9 a, Low magnified AFM image of the 2D seeds and **b,** height profiles along the white lines shown in the AFM image. **c,** Using the AFM profiling analysis, the average height (H_n) of the 2D seeds was measured as 14.4 nm with narrow dispersity.

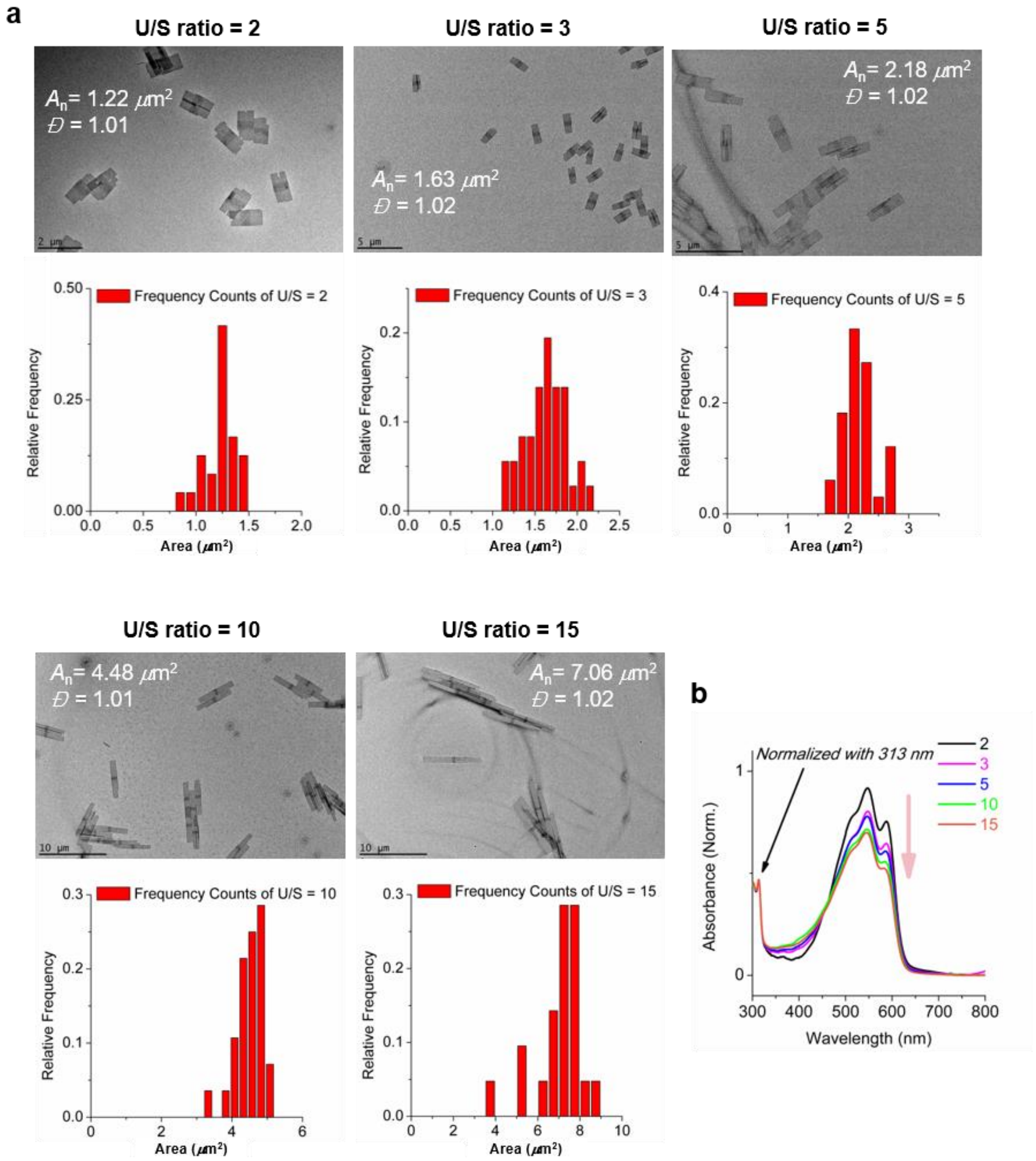

Supplementary Fig. 10 a, Low magnified TEM images and average area (A_n) histograms of the resulting 2D rectangles prepared by living 2D CDSA from seeded-growth of P2$_{10}$ unimers onto 2D seed in 0.03 gL chloroform with various U/S ratios of 2, 3, 5, 10, and 15 (3 weeks aging). Numbers in the images indicate the A_n and its dispersity. **b,** Normalized UV-vis absorbance spectra of resulting 2D rectangles after 3 weeks aging. Due to the concentration limit of UV-vis analysis, the solutions was diluted to 0.05 g/L before analyzing its UV-vis absorbance.

a

U/S ratio	Length (L_n) along (010) direction (μm)	Sigma (σ) (μm)	*Đ*
2	1.54	0.040	1.005
3	2.01	0.132	1.004
5	2.74	0.203	1.01
10	5.75	0.190	1.001
15	8.84	0.350	1.002

b

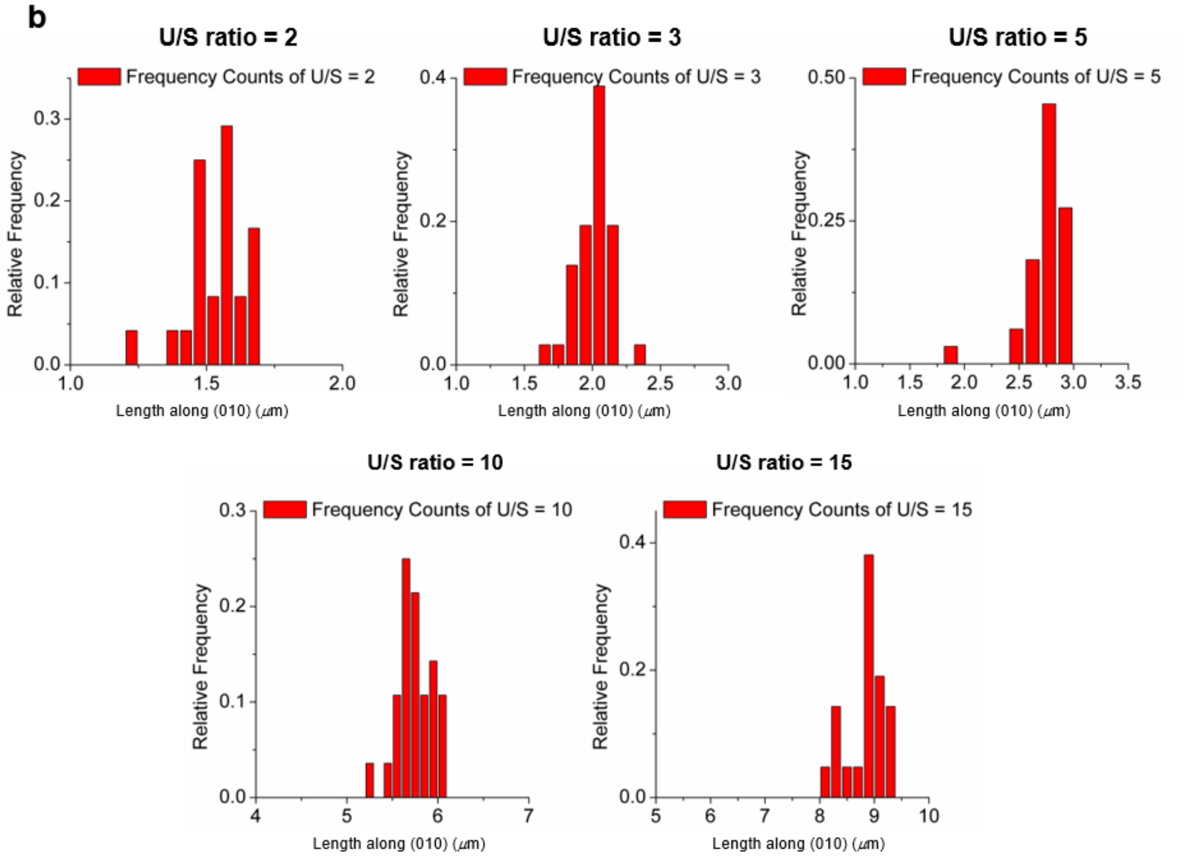

Supplementary Fig. 11 a, A table of the average length (L_n) of the resulting 2D rectangles along the (010) plane of the 2D seeds, and **b,** their contour length histograms.

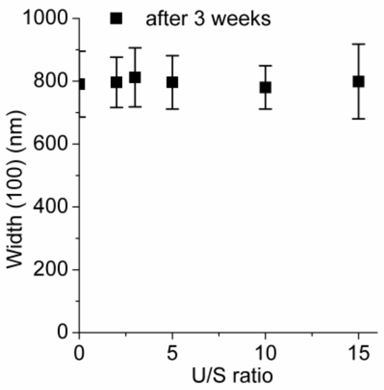

Supplementary Fig. 12 A plot showing the constant average width (W_n) of the resulting 2D rectangles along the (100) plane of the 2D seeds regardless of the U/S ratios. Error bars indicate standard deviations (σ).

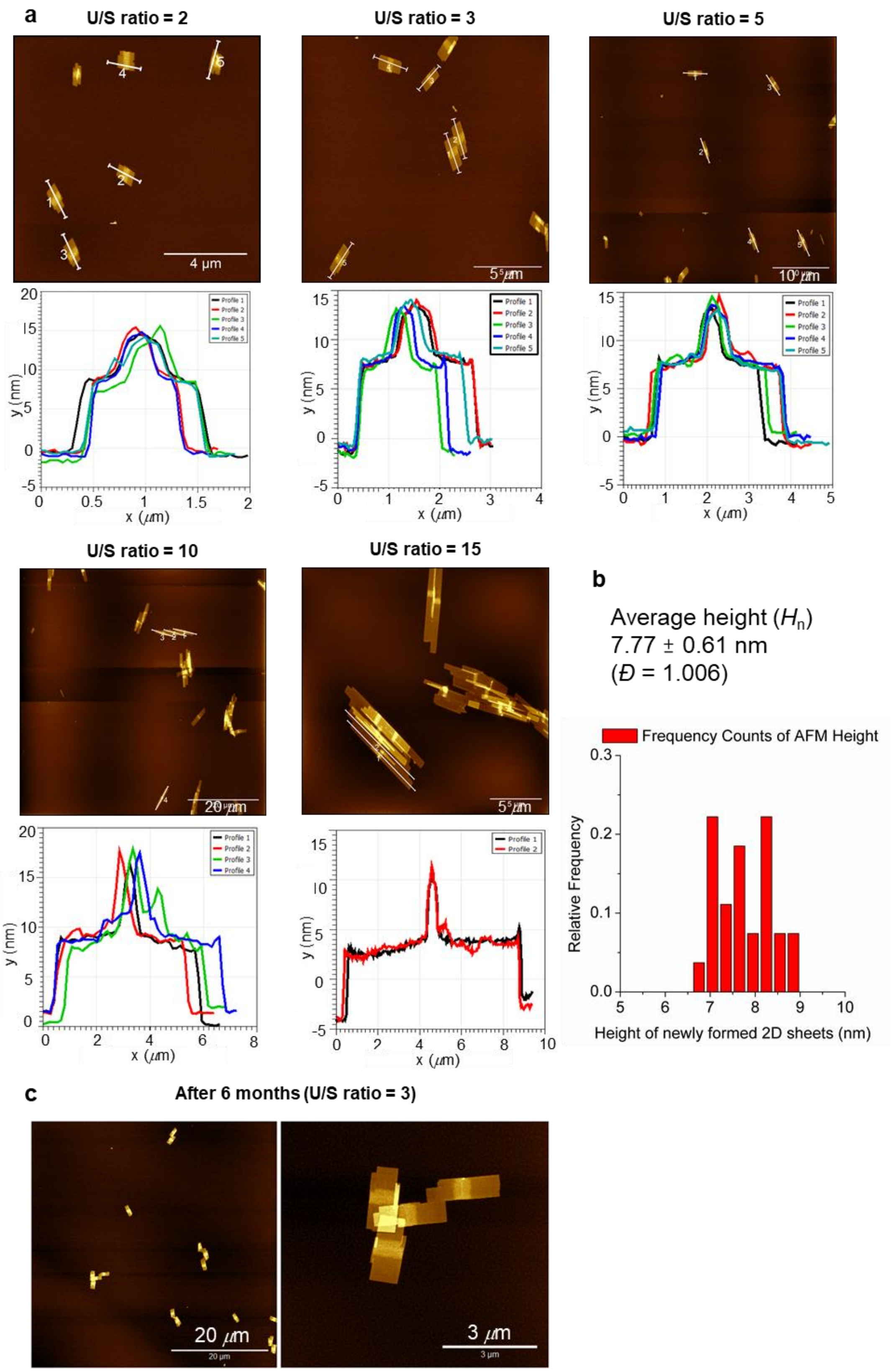

Supplementary Fig. 13 a, Low magnified AFM height images of the resulting 2D rectangles from P2$_{10}$ unimer with various U/S ratios and their height profiles along the white lines shown in the AFM images. The average height (H_n) of the 2D rectangles was calculated as 7.77 ± 0.61 nm. **b,** A height histogram of the 2D rectangles. **c,** AFM images after 6 months at -13 °C aging showing long-term stability of the resulting 2D rectangles.

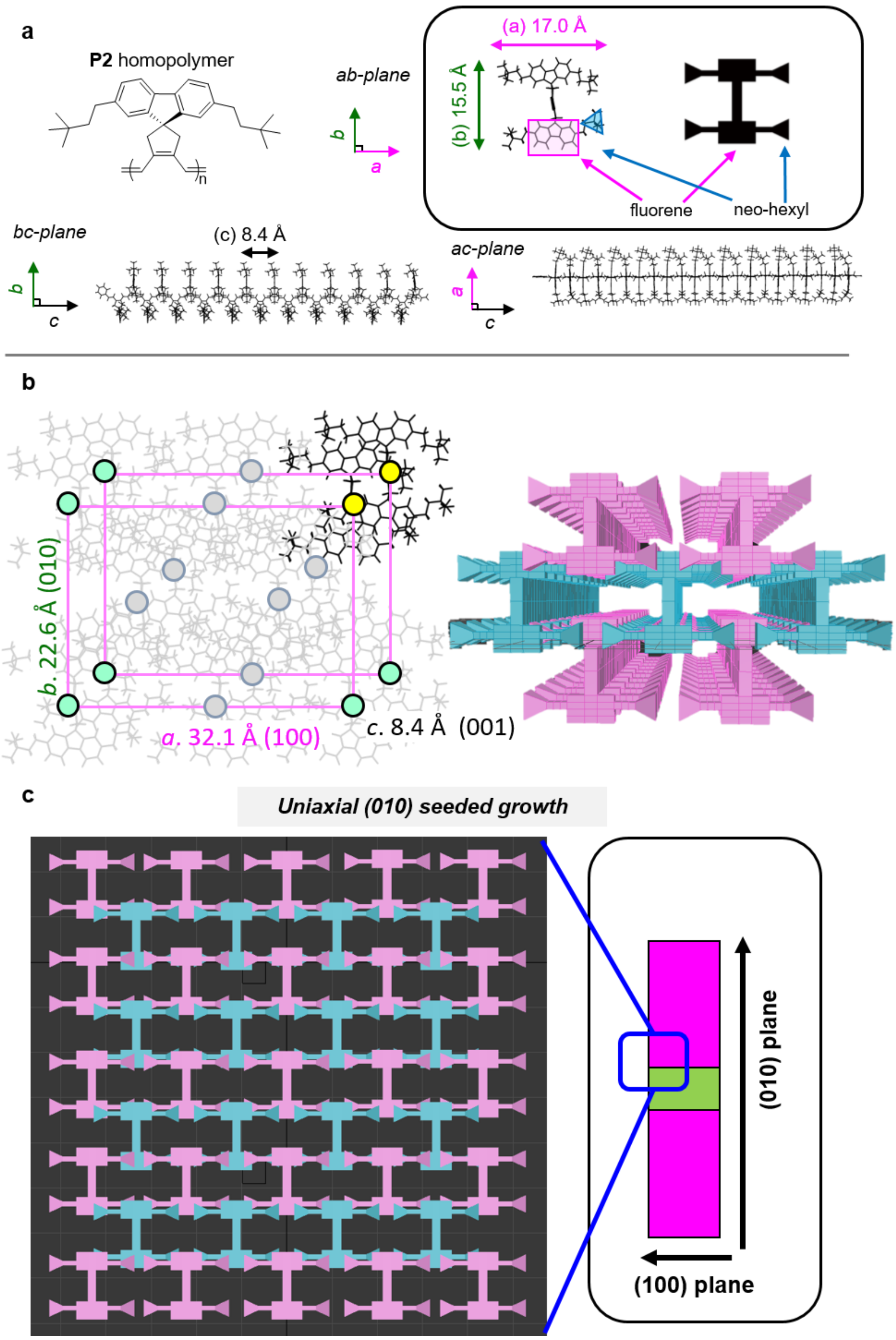

Supplementary Fig. 14 a, The proposed structure of the individual P2 single chain with estimated distance for three axes.[4] **b,** Proposed model of an orthorhombic unit cell of P2 polymer and 3D schematic illustration of 2D arrays of P2 chains on *ab-plane*. The fluorene moieties on the P2 polymer chains are described as rectangles and the neo-hexyl groups as triangles. In detail, the interdigitating slip-stack packing of P2 chains are described as **c,** the low-magnified illustration of the 2D arrangements of P2 chains on *ab-plane*. Based on this 2D illustration, we propose that the neohexyl moiety is exposed along the (100) direction, and the fluorene moiety is exposed along the (010) direction. This difference would probably lead to different surface energy, resulting in the preferential crystallization along one direction.

Time	Length (010)	σ	Length (010)	σ	Length (010)	σ	Length (010)	σ	Length (010)	σ
hours	nm									
	US 2		US 3		US 5		US 10		US 15	
0	438.02	39.2	438.02	39.2	438.02	39.2	438.02	39.2	438.02	39.2
3	--	--	--	--	--	--	568.21	42.38	610.68	48.5
7	--	--	--	--	567.71	42.18	725.6	41.7	817.87	75.78
18	--	--	674.78	125.21	--	--	--	--	--	--
25	627.03	45.51	677.03	45.78	780.16	45.17	1134.9	74.05	1461.08	63.2
48	707.79	69.72	836.36	67.006	966	56.97	1613.6	73.39	2157.49	149.01
70	842.64	42.17	918.41	49.75	1126.76	58.11	2078.4	89.93	2891.19	96.04
101	--	--	1138.08	82.46	1389.47	59.89	2616.5	102.25	3674.31	169.94
122	928.077	136.96	1202.154	62.98	1437.78	104.47	2893.9	104.6	4063.1	198.13
168	--	--	1353.65	66.97	1758.92	185.52	3424.6	177.2	5181.38	356.41
216	--	--	1502.47	80.21	2174.67	718.65	3980.7	226.9	5800.6	358.4
292	1290.04	92.83	1761.54	93.32	--	--	--	--	--	--
336	1406.91	76.37	--	--	2361.78	148.28	4783.4	260.4	7364.61	284.8
411	1418.81	89.97	1876.82	129.94	2663.13	174.9	5350.65	312.7	8317.27	636.5
504	1536.3	105.39	2007.51	131.65	2737.09	203.23	5751.8	189.5	8837.14	349.9

Supplementary Table 1. Statistical Data summary for unimer concentration: Growth kinetic studies on seeded-growth of the resulting 2D rectangles from $P2_{10}$ homopolymer in 0.03/L chloroform with various U/S ratios monitored for 3 weeks at -13 °C. The L_n of 2D seeds was re-measured after dilution in 0.03 g/L chloroform as 438 nm ($Đ$ = 1.01).

- **U/S ratio of 2 at -13 °C**

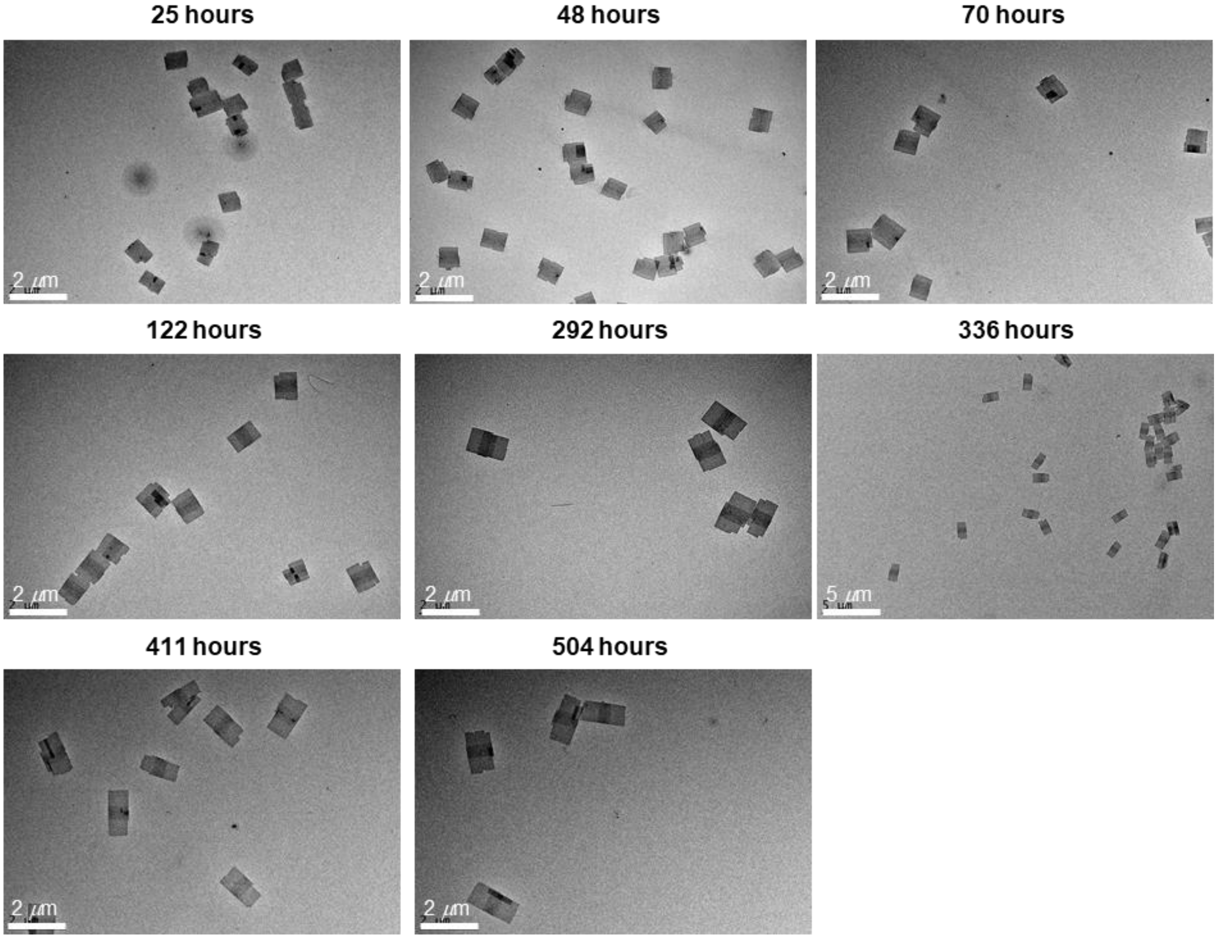

Supplementary Fig. 15 Statistical Data summary for growth kinetic studies with various unimer concentration: TEM images of growing 2D rectangles from $P2_{10}$ over time with a U/S ratio of 2.

- **U/S ratio of 3 at -13 °C**

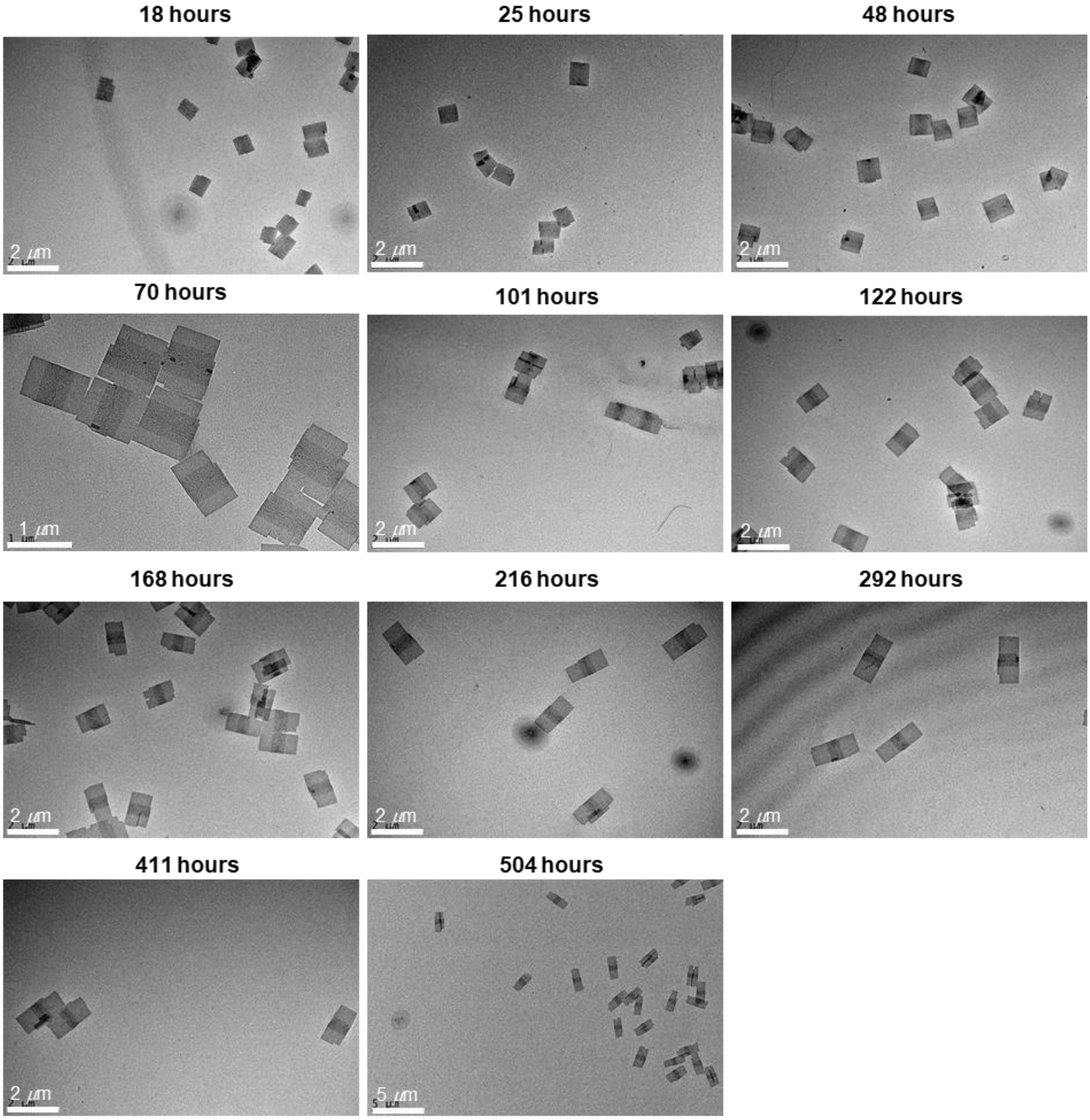

Supplementary Fig. 16 Statistical Data summary for growth kinetic studies with various unimer concentration: TEM images of growing 2D rectangles from $P2_{10}$ over time with a U/S ratio of 3.

- **U/S ratio of 5 at -13 °C**

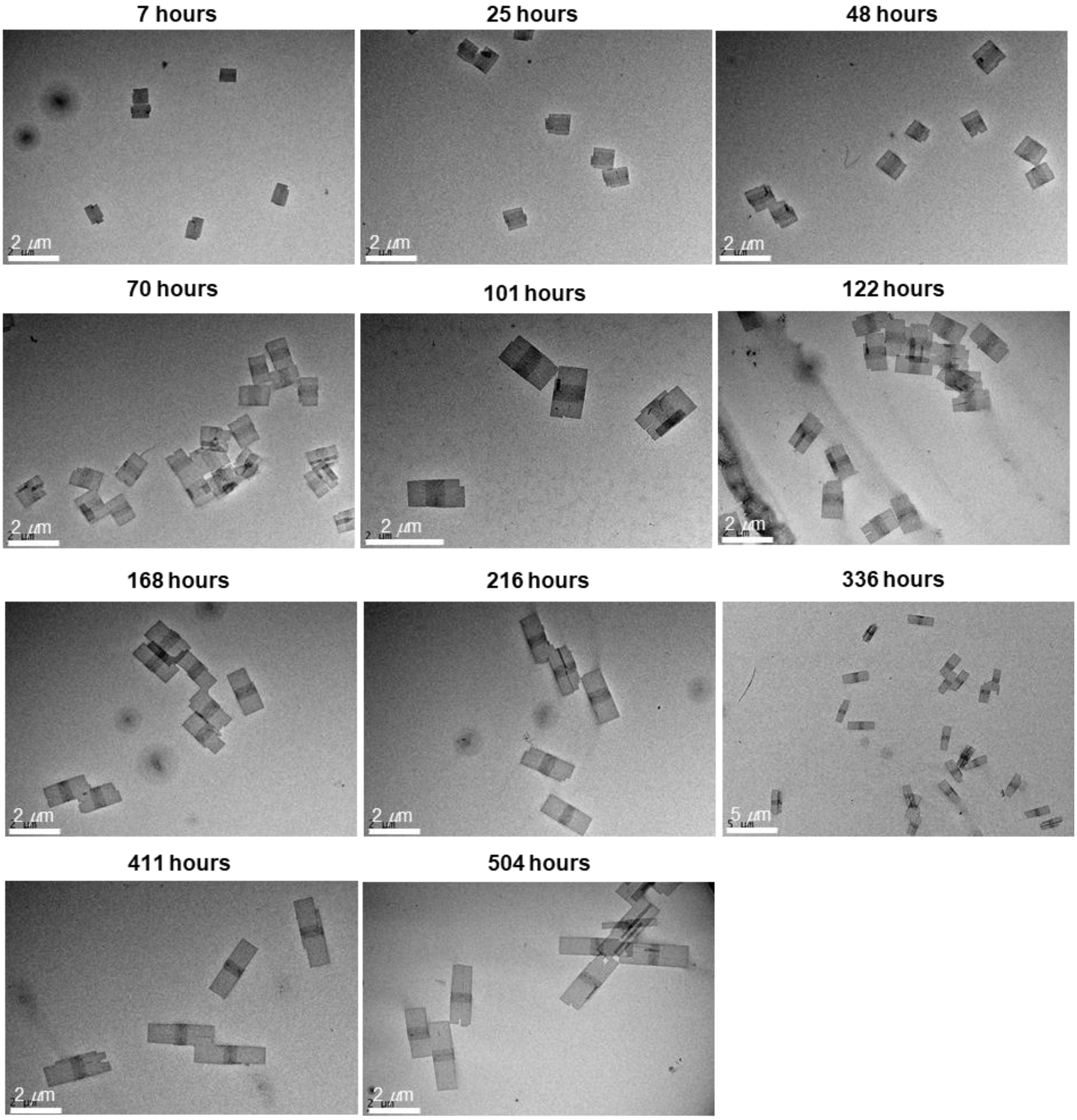

Supplementary Fig. 17 Statistical Data summary for growth kinetic studies with various unimer concentration: TEM images of growing 2D rectangles from $P2_{10}$ over time with a U/S ratio of 5.

- **U/S ratio of 10 at -13 °C**

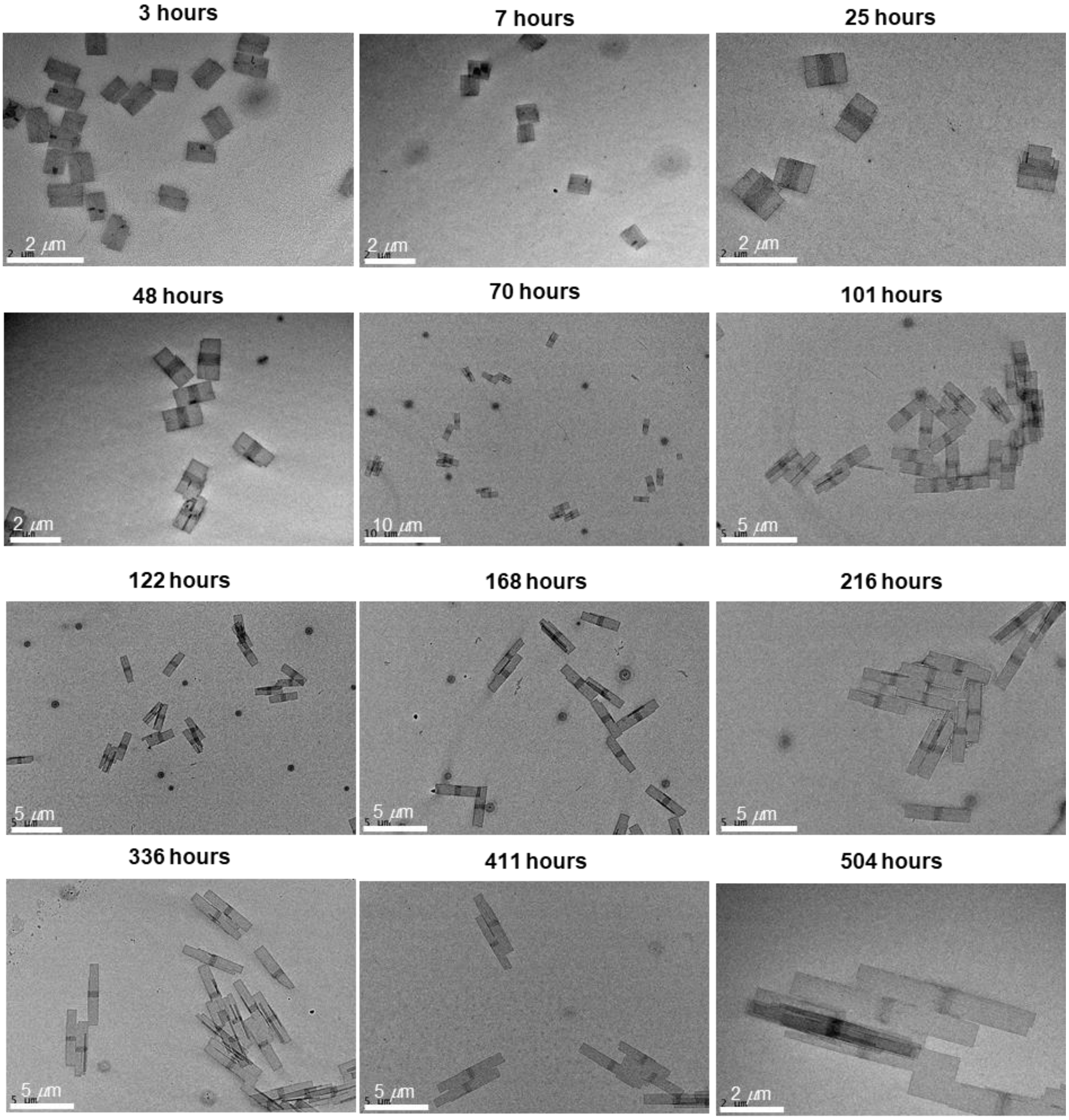

Supplementary Fig. 18 Statistical Data summary for growth kinetic studies with various unimer concentration: TEM images of growing 2D rectangles from $P2_{10}$ over time with a U/S ratio of 10.

- **U/S ratio of 15 at -13 °C**

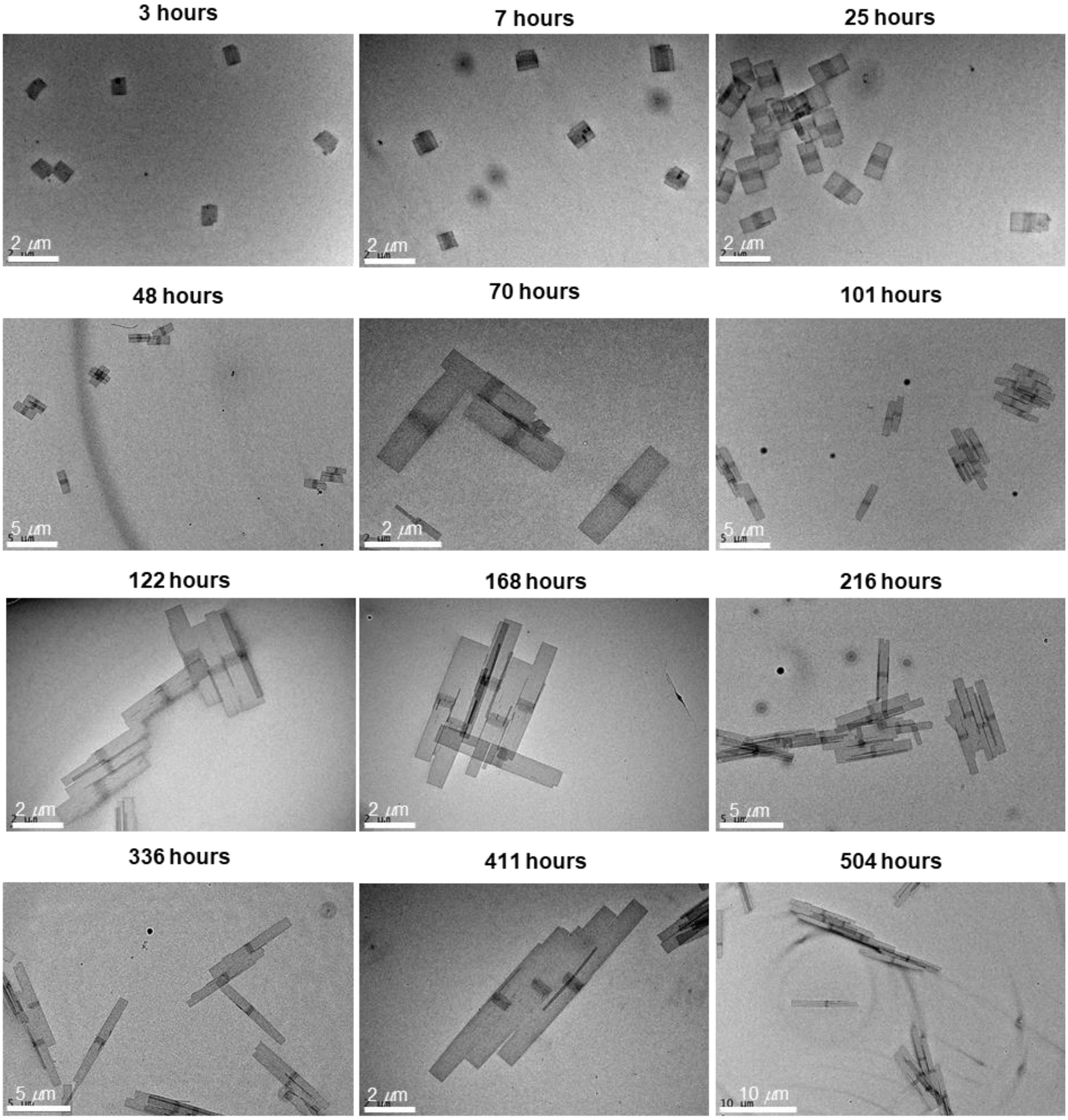

Supplementary Fig. 19 Statistical Data summary for growth kinetic studies with various unimer concentration: TEM images of growing 2D rectangles from $P2_{10}$ over time with a U/S ratio of 15.

$$L(\mathrm{t}) = \frac{1}{N\frac{agg}{L}}\frac{[U]_0}{[S]_0}\left(1 - e^{-2k[S]_0 t}\right) + L_{seed}$$

Equation 1 First-order kinetic function of the seeded-growth of unimer ($[U]_0$ = Initial concentration of unimer solutions, $[S]_0$ = Initial concentration of seed solutions, $N_{agg/L}$ = the linear aggregation number (number of unimer molecules per unit length along the (010) plane), L_{seed} = average length of 2D seeds, L(t) = experimentally determined length by TEM imaging.).[1]

Operation Time	10/12/20 16:45:22
Model	_firstorder (User)
Equation	y = a*(1 - exp(-b*x))+c
Report Status	New Analysis Report
Multi-Data Fit Mode	Independent Fit - Consolidated Report

Input Data

Parameters

		Value	Standard Error
Length (010)	a	1076.51612	43.69654
	b	0.00647	6.06344E-4
	c	438	0
	a	1651.08376	75.76718
	b	0.00513	4.02673E-4
	c	438	0
	a	2466.38286	141.24549
	b	0.00485	4.23721E-4
	c	438	0
	a	5219.57738	304.67537
	b	0.00537	4.61223E-4
	c	438	0
	a	9106.06102	348.14078
	b	0.00444	2.52419E-4
	c	438	0

All datasets were fitted successfully.
Some parameter values were fixed.
Some input data points are missing.

Statistics

	Length (010)				
Number of Points	9	12	12	13	13
Degrees of Freedom	7	10	10	11	11
Reduced Chi-Sqr	0.30823	0.30374	0.50677	0.77126	0.61381
Residual Sum of Squares	2.15761	3.0374	5.06767	8.4839	6.75194
Adj. R-Square	0.9912	0.99325	0.99083	0.99516	0.99724
Fit Status	Succeeded(100)	Succeeded(101)	Succeeded(100)	Succeeded(100)	Succeeded(100)

Supplementary Fig. 20 Kinetic fitting of the data sets of L_n with various U/S ratios (regarding the concentration of unimer, [U]) by the **Equation 1**. Note b (= k') is reported in h^{-1}.

Unimer (kDa)	Aging temperature (°C)	U/S ratio	A (nm)	Error (nm)	k' (h^{-1})	Error (h^{-1})	R^2
5.0	-13	2	1076.5	43.7	6.5×10^{-3}	6.1×10^{-4}	0.991
		3	1651.1	75.8	5.1×10^{-3}	4.0×10^{-4}	0.993
		5	2466.4	141.2	4.9×10^{-3}	4.2×10^{-4}	0.991
		10	5219.6	304.7	5.4×10^{-3}	4.6×10^{-5}	0.995
		15	9106.1	348.1	4.4×10^{-3}	2.5×10^{-5}	0.997

Supplementary Table 2. Kinetic data obtained by fitting of the L_n growth data from **Supplementary Fig. 20** with the **Equation 1**.

$$\frac{rate_i}{rate_j} = \frac{k[U]_i^{\ x}}{k[U]_j^{\ x}} = \left(\frac{[U]_i}{[U]_j}\right)^x$$

$$L_{\infty} - L_{time} = approximates\ to\ L_{final} - L_{time} = constant\ \times\ [\mathrm{U}]_0$$

$$\text{Initial rate} = -\frac{d[U]_0}{dt} = -\frac{d(L_{final} - L_{time})}{dt} = k'[\mathrm{U}]_0^{\ x}$$

$$\log\ (\text{initial rate}) = x\ \times log\ ([U]_0) + constant'$$

Equation 2. Initial reaction rates method for determining reaction order and its application to the length increasement of nanostructures.

a

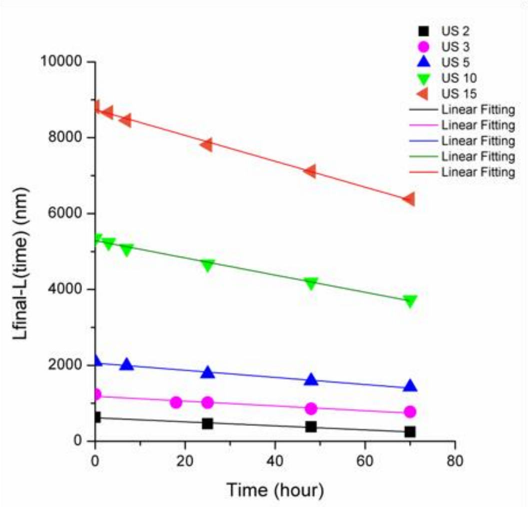

b

Notes

Description	Perform Linear Fitting
User Name	°¹ê
Operation Time	10/12/20 15:21:07
Equation	y = a + b*x
Report Status	New Analysis Report
Weight	No Weighting
Multi-Data Fit Mode	Independent Fit - Consolidated Report

Input Data

Bad Data (missing values) -- Values that are invalid and thus not used in computations

Parameters

		Value	Standard Error
Lfinal-L(time)	Intercept	617.70902	19.46392
	Slope	-5.32347	0.43995
	Intercept	1182.87491	36.31719
	Slope	-6.32158	0.89937
	Intercept	2058.80382	28.09987
	Slope	-9.41346	0.70792
	Intercept	5286.01346	28.33436
	Slope	-22.67536	0.78151
	Intercept	8743.72631	35.38043
	Slope	-34.11208	0.97585

Some input data points are missing.

Statistics

	Lfinal-L(time)				
Number of Points	4	5	5	6	6
Degrees of Freedom	2	3	3	4	4
Residual Sum of Squares	1051.705	7204.0907	5078.59315	9736.63684	15181.28013
Adj. R-Square	0.97979	0.92367	0.97776	0.99409	0.99592

Summary

Supplementary Fig. 21 a, Plots of the approximate to L_{final} – L(time) *versus* time (for 3 days) to obt ain initial growth rate constants. **b,** Kinetic fitting of the initial data sets by the linear equation. All plot s generated straight lines (R^2 = 0.924 – 0.996). The L_{final} value was assumed to be that of 2D recta ngles generated through living 2D CDSA with U/S ratio of 5 at -13 °C after 3 weeks of aging in **Sup plementary Table 1**.

	US 2		US 3		US 5		US 10		US 15	
R^2	0.97979		0.92367		0.97776		0.99409		0.99592	
	Value	Standard Error	Value	Standard Error	Value	Standard Error	Value	Standard Error	Value	Standard Error
Intercept	617.709	19.46392	1182.875	36.31719	2058.804	28.09987	5286.013	28.33436	8743.726	35.38043
Slope (-k(initial))	-5.32347	0.43995	-6.32158	0.89937	-9.41346	0.70792	-22.6754	0.78151	-34.1121	0.97585
log(k(initial))	0.726195		0.800826		0.973749		1.355554		1.532908	
log[U]	-1.22185		-1.04576		-0.82391		-0.52288		-0.34679	

Supplementary Table 3. Kinetics data from **Supplementary Fig. 21b** for fitting of the initial growth d ata using the initial rates method to obtain real reaction order of [U].

Time	Length (010)	σ	Length (010)	σ	Length (010)	σ	Length (010)	σ
hours	nm							
	273 K		263 K		253 K		248 K	
0	438.02	39.24	438.02	39.24	438.02	39.24	438.02	39.24
12	679.56	54.75	763.33	42.88	1099.26	49.227	1109.7	68.52
24	968.46	53.06	1174.378	65.7	1686.78	65.95	1708.61	71.35
36	1154.9	66.35	1408.16	126.88	2110.196	91.62	2346.96	110.16
48	1407.96	93.97	1517.43	98.73	2489.87	143.26	3162.5	233.03
60	1562.13	79.585	1680.72	90.1	2700.986	110.39	3697.6	363.32
72	1655.26	107.44	2056	150.13	3033.444	106.81	4032.4	287.8

Supplementary Table 4. Statistical Data summary for aging temperatures: Growth kinetic studies on seeded-growth of 2D rectangles from $P2_{10}$ in 0.03/L chloroform over 3 days with a U/S ratio of 5 at variable temperatures of 0, -10, -20, and -25 °C.

a

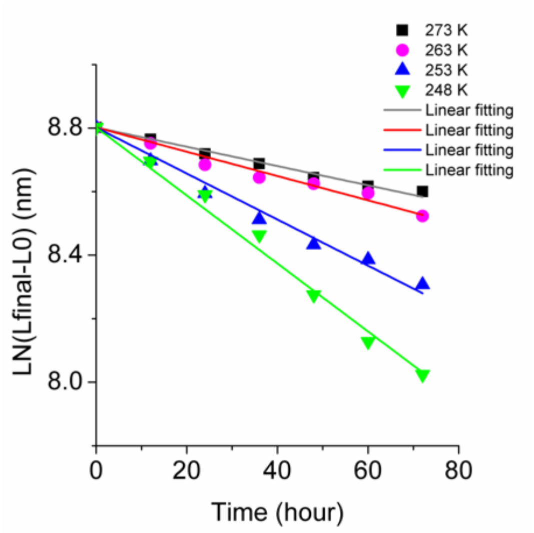

b

Notes

Description	Perform Linear Fitting
User Name	°¢ë
Operation Time	10/10/20 14:23:26
Equation	y = a + b*x
Report Status	New Analysis Report
Weight	No Weighting
Multi-Data Fit Mode	Independent Fit - Consolidated Report

Input Data

Parameters

		Value	Standard Error
LN(Lfinal-L0)	Intercept	8.80207	--
	Slope	-0.00304	8.36198E-5
	Intercept	8.80207	--
	Slope	-0.00382	1.44318E-4
	Intercept	8.80207	--
	Slope	-0.00725	2.20809E-4
	Intercept	8.80207	--
	Slope	-0.01071	2.74525E-4

Statistics

	LN(Lfinal-L0)			
Number of Points	7	7	7	7
Degrees of Freedom	6	6	6	6
Residual Sum of Squares	5.49761E-4	0.00164	0.00383	0.00593
Adj. R-Square	1	1	0.99999	0.99999

Summary

	Intercept		Slope		Statistics
	Value	Error	Value	Error	Adj. R-Square
LN(Lfinal-L0)	8.80207	--	-0.00304	8.36198E-5	1
	8.80207	--	-0.00382	1.44318E-4	1
	8.80207	--	-0.00725	2.20809E-4	0.99999
	8.80207	--	-0.01071	2.74525E-4	0.99999

Supplementary Fig. 22 a, Plots of the approximate to ln(L_{final} – L(time)) *versus* time in **Supplementary Table 4** to obtain initial growth rate constants at various aging temperatures **b,** by fitting into the first-order function in **Equation 1**. Note: b (= -k') was reported in h^{-1}. The L_{final} value was assumed to be that of 2D rectangles generated through living 2D CDSA with U/S ratio of 5 at -13 °C after 3 weeks of aging in **Supplementary Table 1**.

$$\ln\frac{k}{T} = \frac{-\triangle H^{\ddagger}}{R}\frac{1}{T} + ln\frac{k_B}{h} + \frac{\Delta S^{\ddagger}}{R}$$

Equation 3 Eyring equation to determine activation enthalphy and entropy. k = reaction rate constant, $\Delta H^{\ddagger}$ = enthalpy of activation, R = gas constant, T = absolute temparature, k_B = Blotzmann constant, h = Planck's constant, and $\Delta S^{\ddagger}$ = entropy of activation.

	273 K		263 K		253 K		248 K	
R^2	1		1		0.999		0.999	
	value	σ	value	σ	value	σ	value	σ
Intercept	8.80207	--	8.80207	--	8.80207	--	8.80207	--
Slope (b)	-0.00304	8.36E-05	-0.00382	1.44E-04	-0.00725	2.21E-04	-0.01071	2.75E-04
k' (-b/2[s0])	0.050667		0.063667		0.120833		0.1785	
k'/T	0.000186		0.000242		0.000478		0.00072	
ln(k'/T)	-8.59196		-8.32625		-7.64673		-7.2366	
1/T	0.003663		0.003802		0.003953		0.004032	
1000/T	3.663004		3.802281		3.952569		4.032258	

Supplementary Table 5 Kinetics data from **Supplementary Fig. 22b** to determine the activation enthalpy and entropy using Eyring equations in **Equation 3**.

a

Notes

Description	Perform Linear Fitting
User Name	°ŧë
Operation Time	10/11/20 16:24:07
Equation	y = a + b*x
Report Status	New Analysis Report
Weight	No Weighting

Input Data

Bad Data (missing values) -- Values that are invalid and thus not used in computations

	Rows	Y Data	X Data
LN(k/T)	1	--	--

Parameters

		Value	Standard Error
LN(k/T)	Intercept	-22.43693	1.94365
	Slope	3.81014	0.5032

Some input data points are missing.

Statistics

	LN(k/T)
Number of Points	4
Degrees of Freedom	2
Residual Sum of Squares	0.04082
Adj. R-Square	0.94944

Summary

	Intercept		Slope		Statistics
	Value	Error	Value	Error	Adj. R-Square
LN(k/T)	-22.43693	1.94365	3.81014	0.5032	0.94944

b

Y = 3.81 X – 22.44

$\Delta H^{\ddagger}$ (kJ/mol) = - 31.7
$\Delta S^{\ddagger}$ (J/K•mol) = - 384
$\Delta G^{\ddagger}$ (kJ/mol) = 81.6

R^2 = 0.949

Supplementary Fig. 23 a, Kinetic fitting of the data sets in **Supplementary Table 5** by **Equation 3**. **b,** The activation energy and entropy were negative values of -31.7 kJ/mol and -384J/K•mol with R^2 of 0.949.

Time	Length (010)	σ	Length (010)	σ	Length (010)	σ
	nm		nm		nm	
RT	DP 10 (5.0 kDa)		DP 13 (5.6 kDa)		DP 15 (6.1 kDa)	
0	438.02	39.24	438.02	39.24	438.02	39.24
3	--	--	359.07	52.89	435.2	52.22
7	--	--	--	--	510.91	47.94
18	420.08	46.89	515.34	67.66	735.14	59.87
24	--	--	598.45	131.1	840.55	74.46
48	414.37	50.78	740.68	67.15	1164	84.2
72	--	--	--	--	1438.6	99.78
90	--	--	1151.72	81.38	--	--
120	--	--	--	--	1889.49	163.3
135	--	--	1363.17	92.9	--	--
168	568.7	45.6	--	--	2014.65	154.2
194	--	--	1521.3	158.26	--	--
288	717.9	58.83	1592.3	108.14	--	--
336	--	--	1638.8	183.8	--	--
384	--	--		--	2454.71	229.5
672	1170.42	393.54	--	--	--	--

Supplementary Table 6. Statistical Data summary for various molecular weights of P2 unimers: Growth kinetic studies on seeded-growth of 2D rectangles from $P2_n$ with 2D seeds in 0.03 g/L chloroform over 3 weeks with U/S ratio of 5 at 25 °C.

Notes

Description	Perform Linear Fitting
User Name	°¢ë
Operation Time	10/11/20 15:54:10
Equation	y = a + b*x
Report Status	New Analysis Report
Weight	No Weighting
Multi-Data Fit Mode	Independent Fit - Consolidated Report

Input Data

	Input X Data Source	Input Y Data Source	Range
	[Book15]Sheet1!Time	[Book15]Sheet1!LN(Lfinal-L0)	[1:10]
	[Book15]Sheet1!Time	[Book15]Sheet1!LN(Lfinal-L0)	[1:10]
	[Book15]Sheet1!Time	[Book15]Sheet1!LN(Lfinal-L0)	[1:10]

Bad Data (missing values) -- Values that are invalid and thus not used in computations

Parameters

		Value	Standard Error
LN(Lfinal-L0)	Intercept	8.5413	--
	Slope	-1.31554E-4	4.25517E-5
	Intercept	8.5413	--
	Slope	-0.0015	5.91579E-5
	Intercept	8.5413	--
	Slope	-0.00251	1.39895E-4

Some input data points are missing.

Statistics

	LN(Lfinal-L0)		
Number of Points	4	6	8
Degrees of Freedom	3	5	7
Residual Sum of Squares	1.67586E-4	5.16706E-4	0.007
Adj. R-Square	1	1	0.99999

Supplementary Fig. 24 Kinetic fitting of the data sets from **Supplementary Table 6** by using the **Equation 1**. Note: b (= -k') is reported in h^{-1}. The L_{final} value was assumed to be that of 2D rectangles from $P2_{10}$ unimers generated through living 2D CDSA at -13 °C with U/S ratio of 5 after 3 weeks of aging in **Supplementary Table 1**.

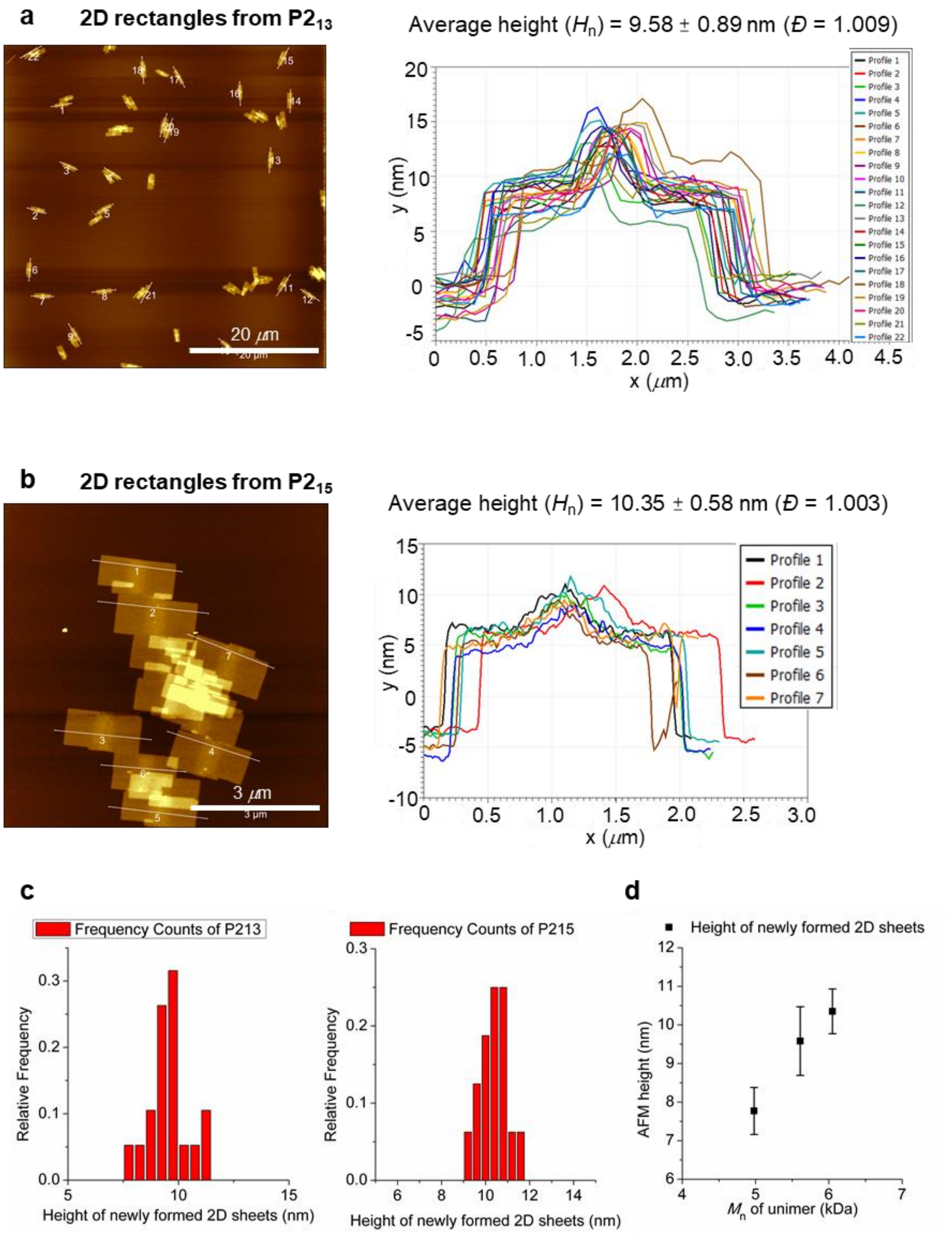

Supplementary Fig. 25 a-b, AFM images of 2D rectangles from **a,** P2$_{13}$ unimers (9.6 ± 0.9 nm) and **b,** P2$_{15}$ unimers (10.4 ± 0.6 nm), and their height profiles along the white lines shown in the AFM images. **c,** Their height histograms obtained by AFM imaging. **d,** A plot of H_n *versus* M_n of P2 unimers showing linear dependence.

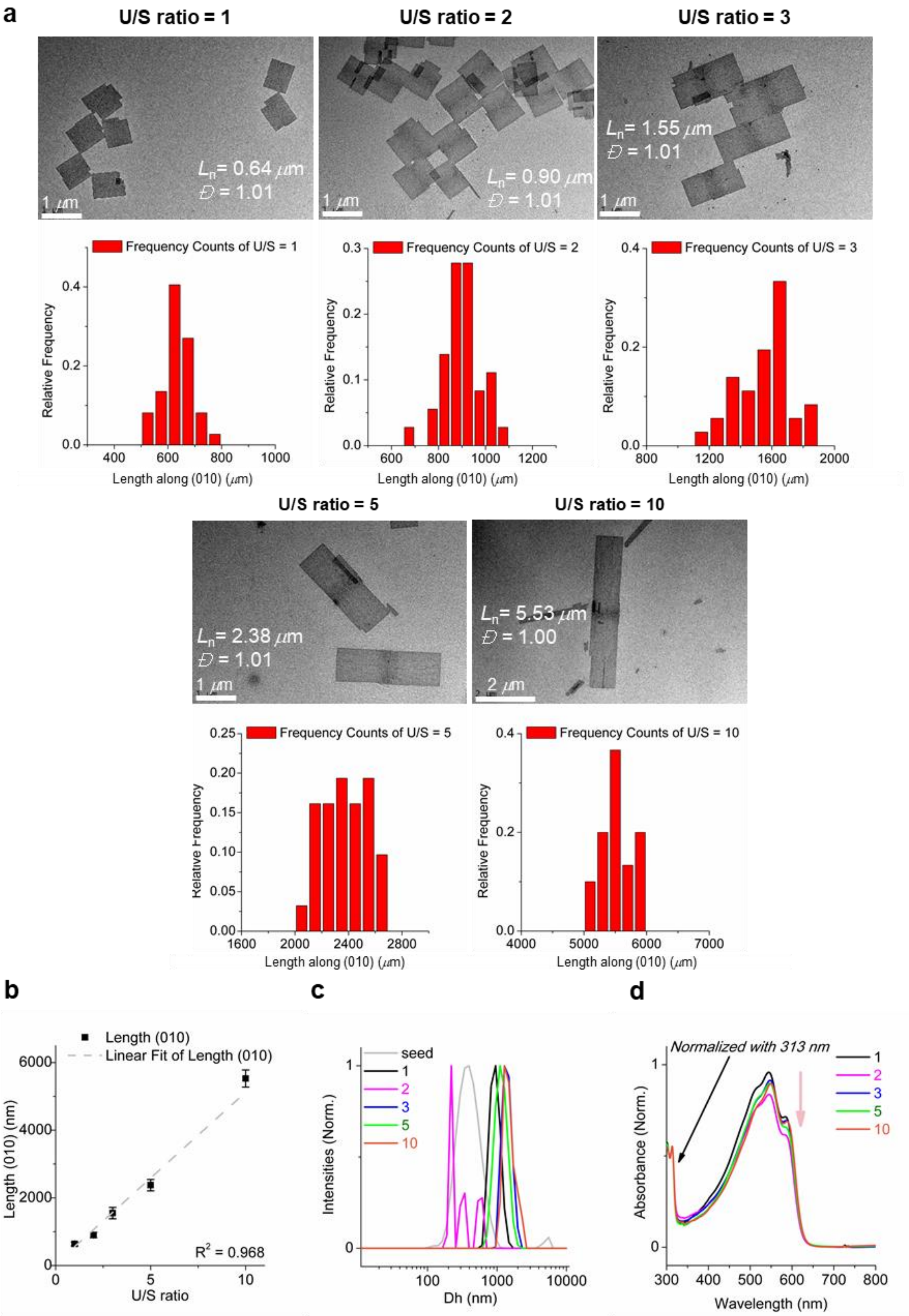

Supplementary Fig. 26 a, Low magnified TEM images and contour length histograms of the resulting 2D rectangles prepared by living 2D CDSA from P2$_{13}$ unimers in seed 0.03 gL chloroform solution aft er 3 weeks aging at 25 °C (U/S ratios = 1, 2, 3, 5, and 10). Numbers in TEM images indicate the L_n and its length dispersity. **b,** A plot of the L_n *versus* U/S ratio showing the livingness of 2D CDSA. **c,** D LS profiles and **d,** Normalized UV-vis absorbance spectra of the resulting 2D nanosheets with 313 n m absorption from fluorene moiety.

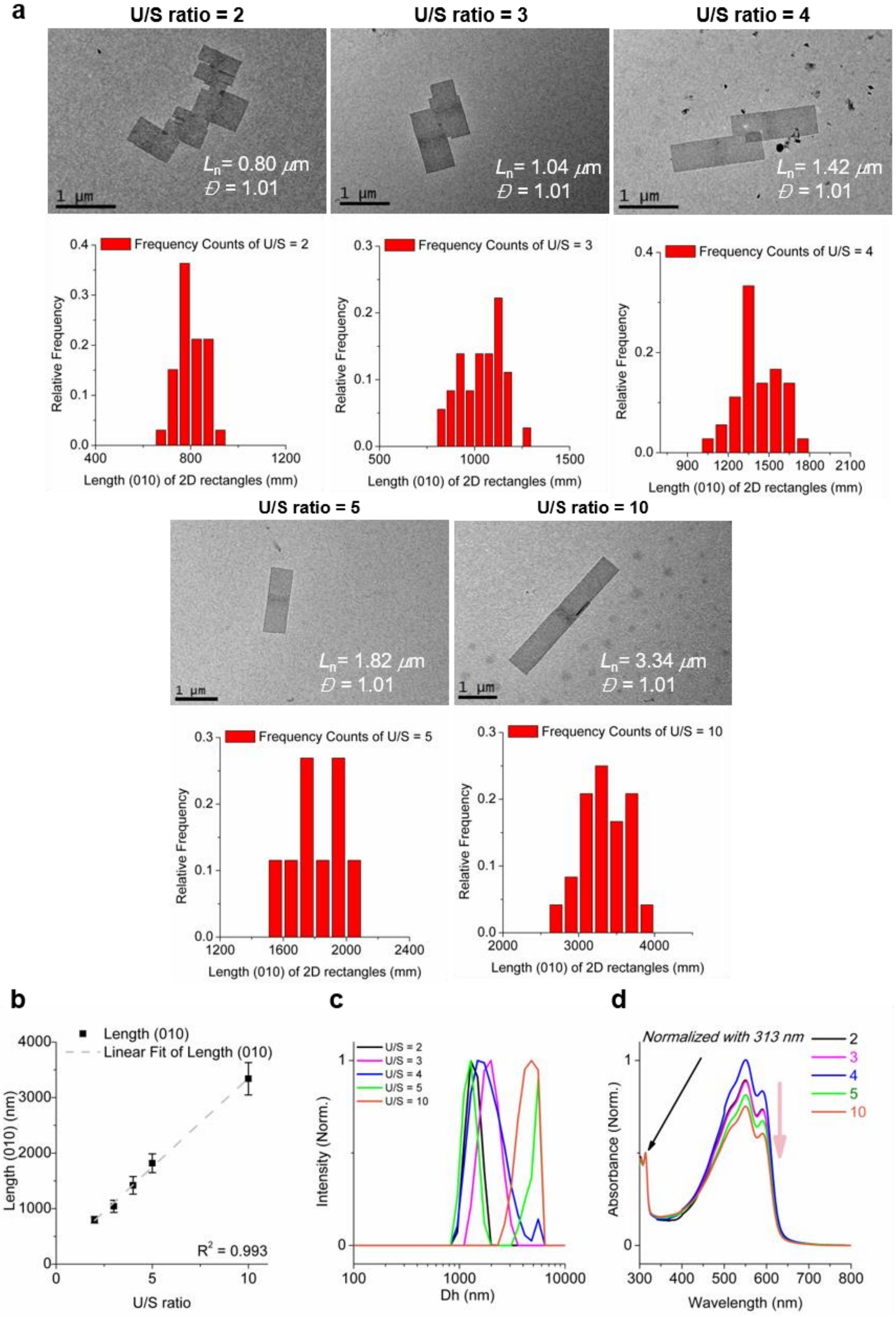

Supplementary Fig. 27 a, Low magnified TEM images and contour length histograms of the resulting 2D rectangles prepared by living 2D CDSA from $P2_{15}$ unimers in seed 0.05 gL chloroform solution aft er 2 weeks aging at 25 °C (U/S ratios = 2, 3, 4, 5, and 10). Numbers in TEM images indicate the L_n and its length dispersity. **b,** A plot of the L_n *versus* U/S ratio showing the livingness of 2D CDSA. **c,** D LS profiles and **d,** Normalized UV-vis absorbance spectra of the resulting 2D nanosheets with 313 n m absorption from fluorene moiety.

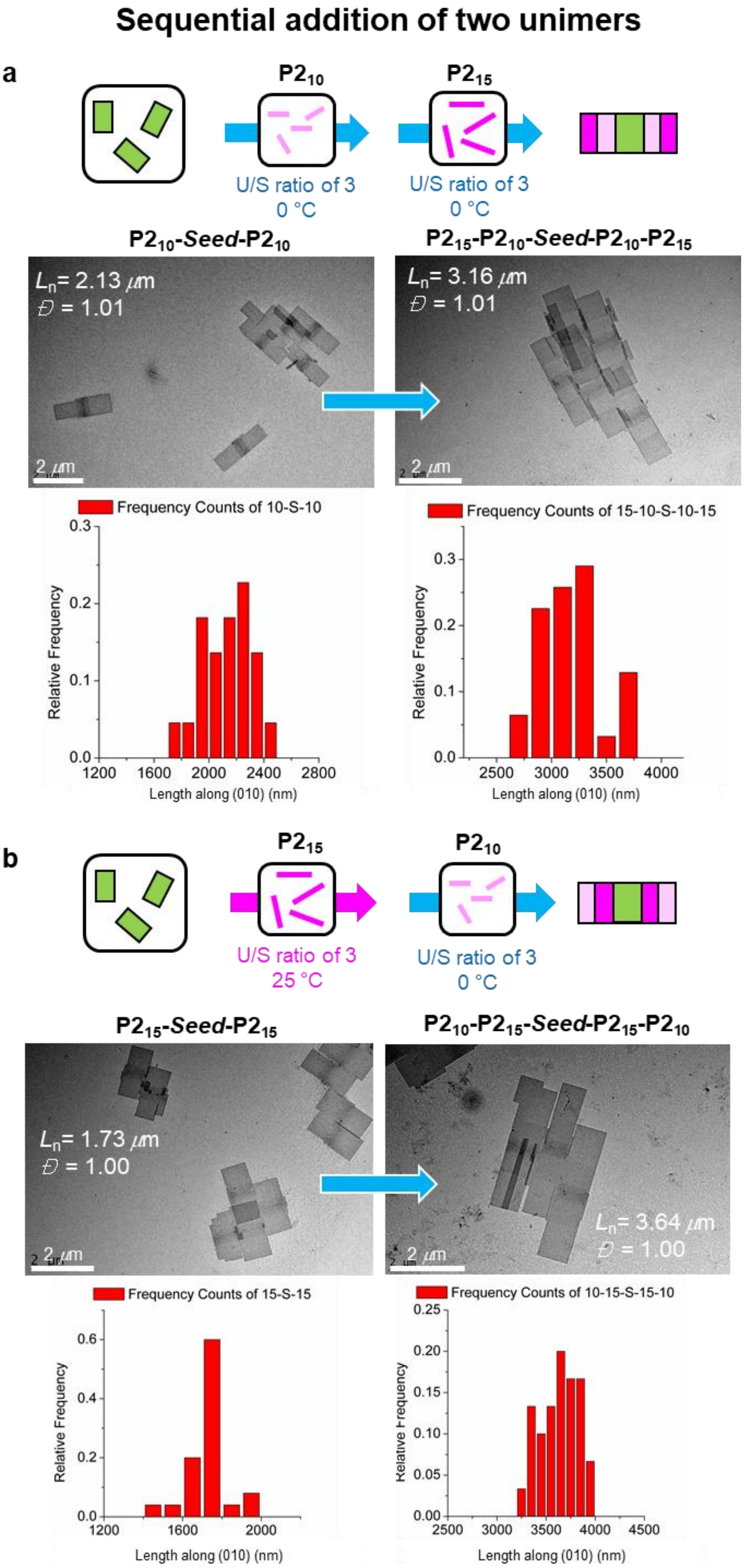

Supplementary Fig. 28 An illustration of formation of penta-BCM by sequential addition of two kinds of unimer by changing the addition order of two unimers. **a,** TEM images and their length histograms of B(P2$_{10}$)-S(seed)-B tri-BCM and A(P2$_{15}$)-B(P2$_{10}$)-S(seed)-B-A penta-BCM prepared by living 2D CDSA at 0 °C. **b,** TEM images and their length histograms of A(P2$_{15}$)-S(seed)-A tri-BCM and B(P2$_{10}$)-A(P2$_{15}$)-S(seed)-A-B penta-BCM prepared by living 2D CDSA at variable temperature (25 °C aging for the first P2$_{15}$ unimers to reduce self-nucleation of P2$_{15}$ unimers and 0 °C aging for the second P2$_{10}$ unimer). Numbers in TEM images indicate the L_n and its length dispersity".

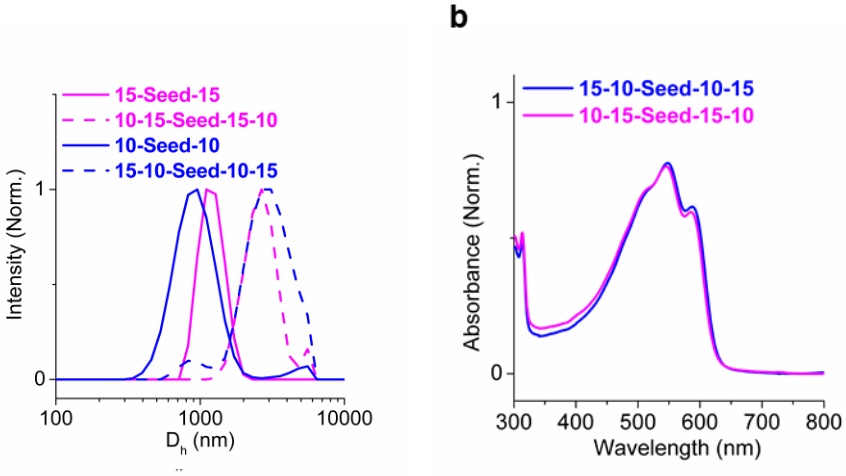

Supplementary Fig. 29 a, DLS profiles and **b,** UV-vis spectra of two types of tri-BCM and penta-BCM prepared by chaning the addition order of two unimers.

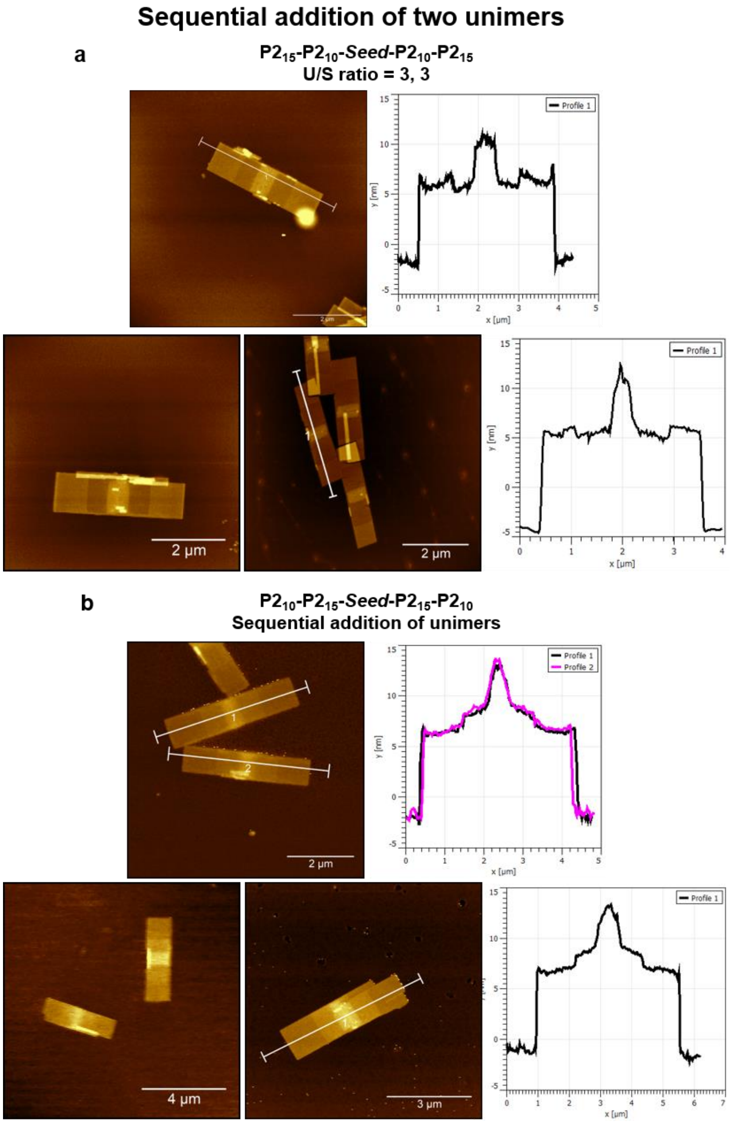

Supplementary Fig. 30 AFM images of **a,** A(P2$_{15}$)-B(P2$_{10}$)-S(seed)-B-A penta-BCM and **b,** B(P2$_{10}$)-A(P2$_{15}$)-S(seed)-A-B penta-BCM prepared by the sequential addition of two unimers with U/S ratios of 3 and their height profiles along the white lines shown in the AFM images. Both penta-BCMs clearly sh ow the height distinction in the height profiles of 2D nanosheets.

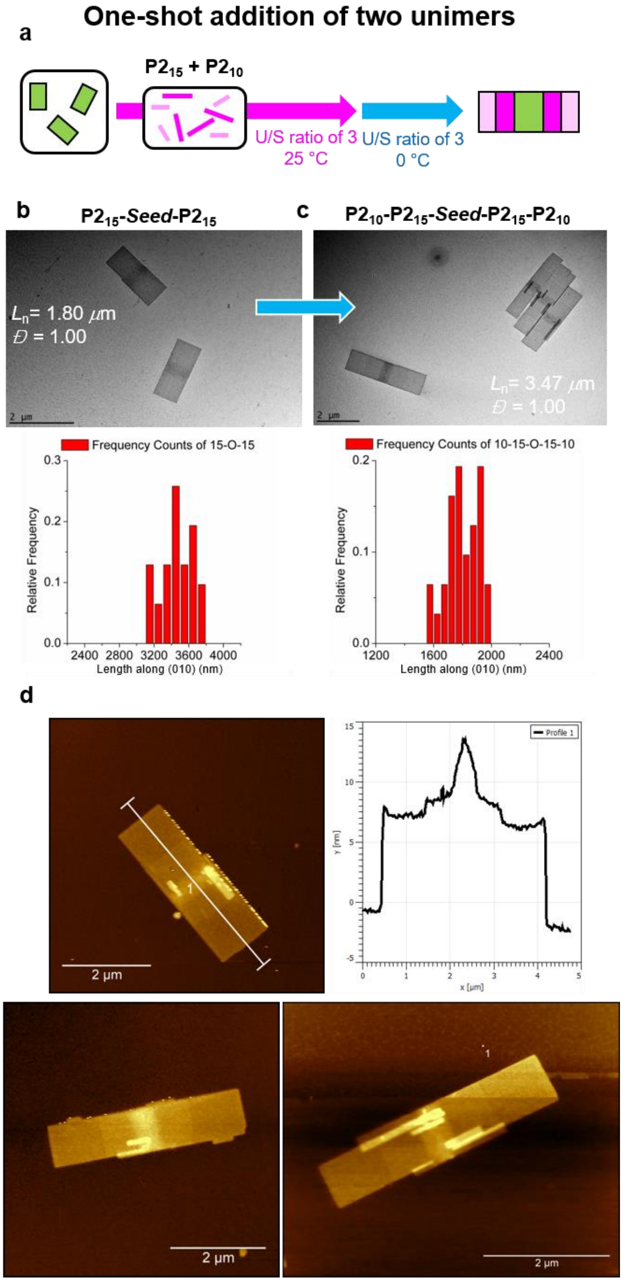

Supplementary Fig. 31 a, An illustration of penta-BCM formation by simple one-shot addition of two kinds of unimer at the same time with each U/S ratio of 3 at 25 °C. **b,** TEM images and histograms of lengths of A($P2_{15}$)-S(seed)-A tri-BCM after preferential assembly of $P2_{15}$ unimers at 25 °C. **c,** B($P2_{10}$)-A($P2_{15}$)-S(seed)-A-B penta-BCM prepared by lowering the aging temperature to 0 °C, resulting the self-assembly of shorter $P2_{10}$. Numbers in TEM images indicate the L_n along and its length dispersity. **d,** AFM images of the B-A-S-A-B type of penta-BCM and height profile along the white line shown in the AFM images.

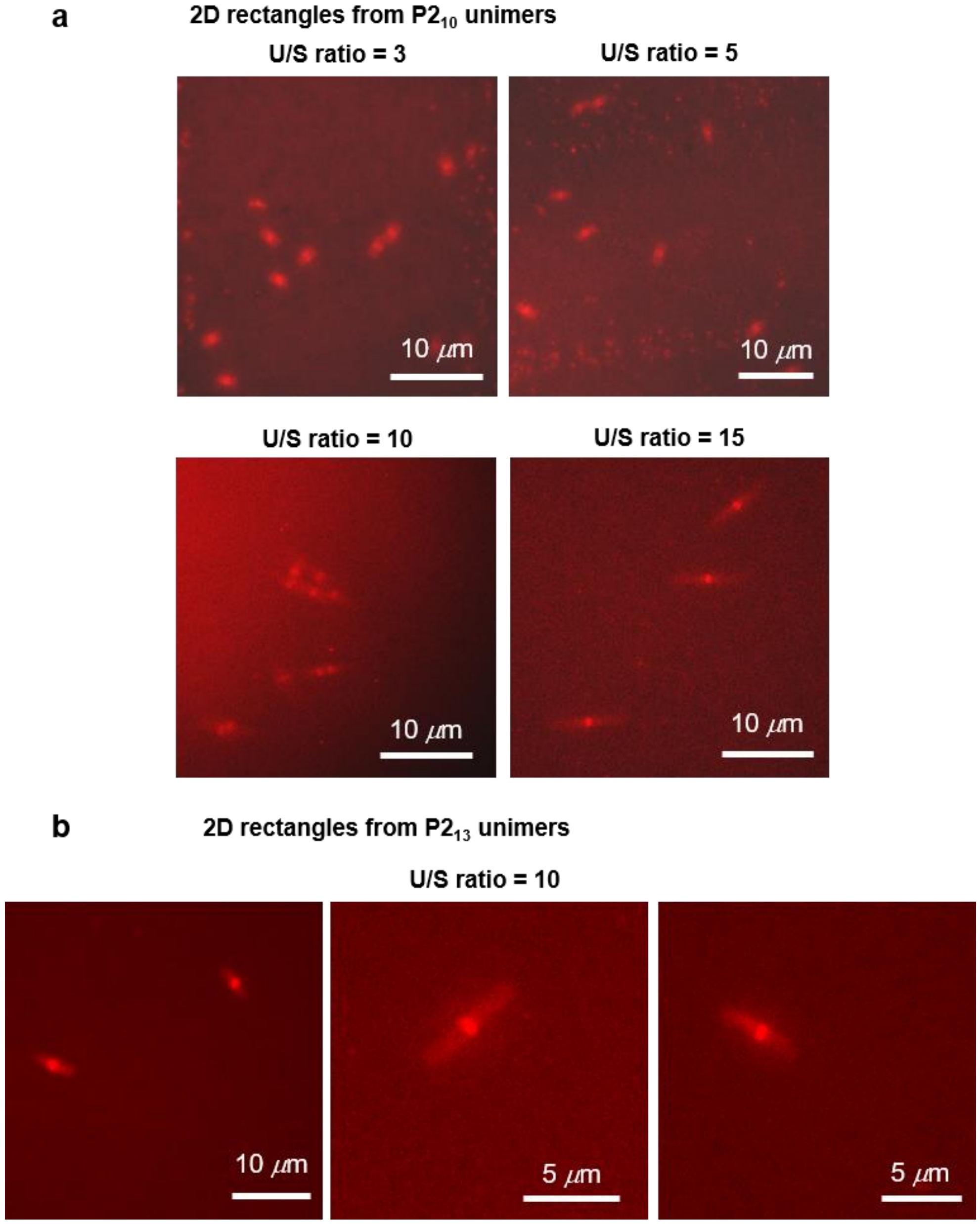

Supplementary Fig. 32 a, Low-magnified fluroescent images of resulting 2D rectangles from P2$_{10}$ ho mopolymer as a unimer with various U/S ratios in dried samples. **b,** Fluorescent images of solution s amples of the length controlled 2D rectangles from P2$_{13}$ unimers with U/S ratio of 10. Dried samples on glass were prepared by spin-coating the drops of the polymer solutions, and visualized by super-r esolution structured illumination microscopy (SR-SIM) using various excitation wavelengths (488 nm an d 561 nm excitation).

4. Supplementary References

1 Boott, C. E. *et al.* Probing the Growth Kinetics for the Formation of Uniform 1D Block Copolymer Nanoparticles by Living Crystallization-Driven Self-Assembly. *ACS Nano* **12**, 8920–8933 (2018).

2 Kang, E.-H. *et al.* Ultrafast Cyclopolymerization for Polyene Synthesis: Living Polymerization to Dendronized Polymers. *J. Am. Chem. Soc.* **133**, 11904–11907 (2011).

3 Yang, S. *et al.* Morphologically Tunable Square and Rectangular Nanosheets of a Simple Conjugated Homopolymer by Changing Solvents. *J. Am. Chem. Soc.* **141**, 19138–19143 (2019).

4 Yang, S. *et al.* Direct Formation of Large-Area 2D Nanosheets from Fluorescent Semiconducting Homopolymer with Orthorhombic Crystalline Orientation. *J. Am. Chem. Soc.* **139**, 3082–3088 (2017).

5 Yang, S. *et al.* Rapid formation and real-time observation of micron-sized conjugated nanofibers with tunable lengths and widths in 20 minutes by living crystallization-driven self-assembly. *Chem. Sci.* **11**, 8416–8424 (2020).
