# Supplementary material for: Semi-conducting 2D rectangles with tunable length via uniaxial living crystallization-driven self-assembly of homopolymer
Source: Nat Commun. 2021 May 10;12:2602. doi: 10.1038/s41467-021-22879-6 (PMC8110585; doi:10.1038/s41467-021-22879-6)
Supplement: Supplementary file 3 — Description of Additional Supplementary Files [file 41467_2021_22879_MOESM3_ESM.pdf]

Description of Additional Supplementary Files

File Name: Supplementary Movie 1

Description: This movie described the dynamics movement of the resulting 2D rectangles prepared by living 2D CDSA of P213 with U/S ratio of 10 in Supplementary Fig. 26. The original video rate was 10 fps (10 frames per second).
